# Impacts of School Nutrition Interventions on the Nutritional Status of School-Aged Children in Asia: A Systematic Review and Meta-Analysis

**DOI:** 10.3390/nu14030589

**Published:** 2022-01-28

**Authors:** Suladda Pongutta, Omotomilola Ajetunmobi, Calum Davey, Elaine Ferguson, Leesa Lin

**Affiliations:** 1 International Health Policy Programme, Tiwanon Rd., Muang, Nonthaburi 11000, Thailand; 2 London School of Hygiene and Tropical Medicine, Keppel St., Bloomsbury, London WC1E 7HT, UK; tomi.ajetunmobi@lshtm.ac.uk (O.A.); calum.davey@lshtm.ac.uk (C.D.); elaine.ferguson@lshtm.ac.uk (E.F.); leesa.lin@lshtm.ac.uk (L.L.); 3 Laboratory of Data Discovery for Health (D24H), Hong Kong Science Park, Hong Kong, China

**Keywords:** school nutrition interventions, school-aged children, nutritional status, obesity, Asia

## Abstract

This review aims to describe school nutrition interventions implemented in Asia and quantify their effects on school-aged children’s nutritional status. We searched Web of Science, Embase, Ovid MEDLINE, Global Health, Econlit, APA PsycInfo, and Social Policy and Practice for English articles published from January 2000 to January 2021. We quantified the pooled effects of the interventions on the changes in body mass index (BMI) and body mass index z score (BAZ), overall and by type of intervention. In total, 28 articles were included for this review, of which 20 articles were multi-component interventions. Twenty-seven articles were childhood obesity studies and were included for meta-analysis. Overall, school nutrition interventions reduced school-aged children’s BMI and BAZ. Multi-component interventions reduced the children’s BMI and BAZ, whereas physical activity interventions reduced only BMI and nutrition education did not change BMI or BAZ. Overweight/obesity reduction interventions provided a larger effect than prevention interventions. Parental involvement and a healthy food provision did not strengthen school nutrition interventions, which may be due to an inadequate degree of implementation. These results suggested that school nutrition interventions should employ a holistic multi-component approach and ensure adequate stakeholder engagement as well as implementation to maximise the effects.

## 1. Introduction

Malnutrition covers various health conditions, including stunting, wasting, underweight, micronutrient deficiencies, overweight, obesity, or diet-related non-communicable diseases [1]. Malnutrition is a leading risk factor for global disability adjusted life years (DALYs), of which children are most affected [2]. The regional trends in malnutrition among children aged 5–19 years are diverse. A four-decade trend (from 1975 to 2016) showed that overnutrition in high-income countries has been stable, while increasing sharply in Asian countries [3]. In addition, overnutrition is the predominant form of malnutrition in high-income countries and some Oceania countries, while a ‘double burden’ of malnutrition—both under- and overnutrition—is prevalent in Asia and Africa. Malnutrition prevents children from developing to their full potential [4], which could affect not only health outcomes but also other pillars of sustainable development such as education and income.

School nutrition interventions have been implemented in many countries across the world using various approaches to address malnutrition among children [5,6]. During the last two decades, several systematic reviews assessing the effectiveness of school-based nutrition interventions have been published [7,8,9,10,11,12,13]. These systematic reviews indicated that the effectiveness of the interventions varied by context. Interventions implemented in high-income western countries [7,8,9] and China [10] were effective, especially multi-component interventions. On the other hand, school nutrition interventions implemented in low- and middle-income countries showed inconclusive results [11,12,13]. Few systematic reviews focused on Asian countries except for China. Therefore, it is still obscure whether school nutrition interventions implemented in the Asian contexts were effective and to what extent.

The effectiveness of school nutrition interventions may also vary by school levels (e.g., pre-, primary, and secondary schools) due to different conditions of children’s growth and development, and different food environments in primary and secondary schools [8,14,15,16]. Primary schools are the intermediate level that provide a great opportunity to improve child nutrition because primary schools reach the majority of the young population [17] and impairments resulting from early child malnutrition could be reduced in primary school children [18]. To make the most of this great opportunity, effective primary school interventions should be implemented. To ensure the effectiveness of school nutrition interventions, the guiding evidence on “what works” and “how” is critical [19]. Unfortunately, the current literature does not focus specifically on assessing the effectiveness of school nutrition interventions implemented in primary schools [7,8,9,10,11,12,13].

To date, the effectiveness of primary school nutrition interventions implemented in Asia is still unknown. This missing piece of evidence is crucial for nutrition policy decisions in Asia. This review, therefore, aims to determine the effectiveness of primary school nutrition programmes on reducing any forms of malnutrition among school-aged children in Asian countries.

## 2. Methods

### 2.1. Search Strategies

This systematic review was conducted following the Preferred Reporting Items for Systematic reviews and Meta-analyses Protocols (PRISMA-P) guidelines [20]. It was reregistered in PROSPERO with registration no. CRD42021226176.

The search was carried out in Web of Science, Embase, Ovid MEDLINE, Global Health, Econlit, APA PsycInfo, and Social Policy and Practice using these following search terms: population (student* or kid* or child* or pupil* or youth* or school?age*), outcome (BMI or body mass index or wast* or stunt* or overweight* or obes* or nutrition?status), and intervention (school?based intervention* or school intervention*). English articles published from January 2000 to January 2021 were included.

### 2.2. Eligibility and Quality Assessment

Double screening was conducted independently by two reviewers using the inclusion and exclusion criteria as follows:

- Inclusion criteria: Population (school-aged children), intervention (school-based nutrition interventions for any types of nutritional status implemented in primary schools in Asia), outcome (BMI, BMI z score, overweight, obesity, stunting, wasting), and study design (complete pre-posttest with control study: randomised control trial or cluster randomised control trial or quasi-experiment)
- Exclusion criteria: Population (school-aged children enrolled in secondary schools or multiple school levels), study design (study protocol of pre-posttest with control study.

The quality of randomised and clustered randomised controlled trials was appraised using the Cochrane risk-of-bias tools for randomised trials and cluster randomised trials [21], respectively. The randomised and cluster randomised controlled trials were categorised into low risk of bias, some concerns, and high risk of bias. Quasi-experiments were assessed for their quality using the ROBINS-I tool [22]. The quasi-experiments were classified into four tiers, namely low, moderate, serious, and critical risk of bias.

### 2.3. Data Extraction and Data Analysis

The following data were extracted from the selected articles: basic bibliographic information, sample size, participant characteristics, study’s objective, study design, intervention characteristics, and outcomes. The effects of the interventions on body mass index (BMI) and body mass index z score (BAZ) were calculated from differences in mean changes from pre-intervention to post-intervention between intervention and control groups.

Heterogeneity was measured using I^2^ statistics. The levels of heterogeneity were rated as low (I^2^ = 25%), moderate (I^2^ = 50%) or high (I^2^ = 75%). Funnel plots and the Egger’s test [23] were performed to assess publication bias using STATA version 16.

Random-effects models with inverse variance methods were used to pool the effect estimates. RevMan5.4 [24] was used to estimate the pooled effects, heterogeneity, and sensitivity. Differences with a *p* < 0.05 were considered as significant. Sensitivity analysis was carried out by excluding experimental studies having a high risk of bias and quasi-experimental studies.

Subgroup analysis was performed according to the following characteristics that were pre-specified in the review protocol: components of interventions (single-component versus multi-component, nutrition education versus extra exercise sessions versus multi-component intervention, and having a healthy food provision (i.e., improved school food environment or food boxes by increasing fruits and vegetables or whole grains, decreasing fat/oil and sugar, and restricting fast food availability in and around schools) versus not having a healthy food provision), duration of intervention (<1 year versus ≥1 year), sample size (<1000 students versus ≥1000 students), and engagement of parents (involved parents versus uninvolved parents).

## 3. Results

### 3.1. Results of Screening Process

**Figure 1:**
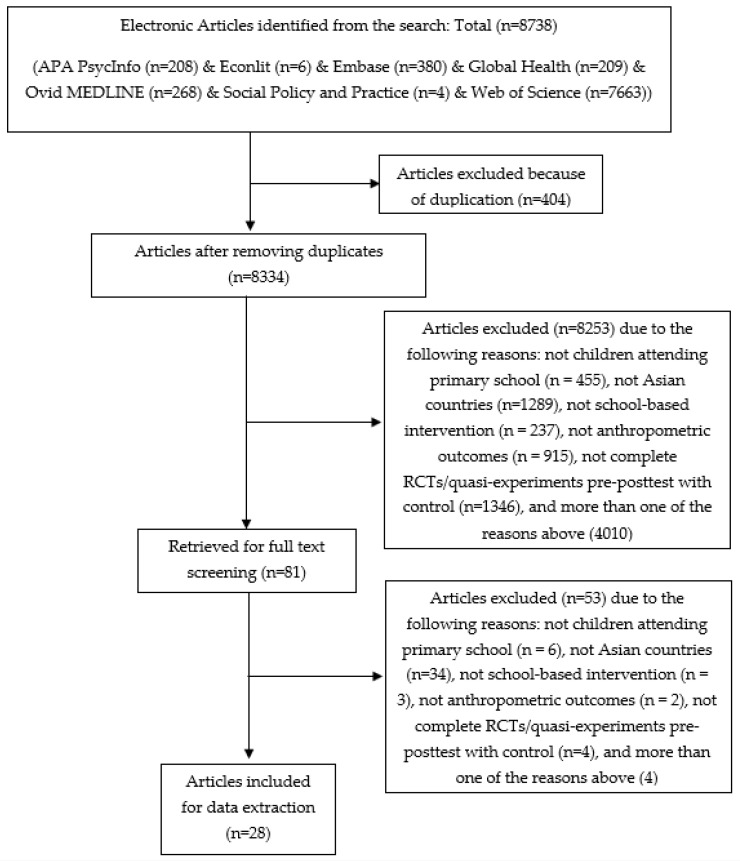
PRISMA diagram of the review process.

The PRISMA diagram of this review is shown in Figure 1. The search yielded 8738 publications. After excluding the duplicates, the titles and abstracts of 8334 publications were screened and 81 articles were retrieved for full-text screening. In total, 28 articles were included for data extraction. The reasons for exclusion were mainly related to study designs (not complete RCTs/quasi-experiments pre-posttest with control) and target populations (not in Asian countries).

### 3.2. Characteristics and Quality of Included Studies

The selected papers were published between 2004 and 2020. These studies included 15 cluster randomized control trials (CRCTs), 10 quasi-experiments, and 3 randomized controlled trials (RCTs), which accounted for 53.6%, 35.7%, and 10.7%, respectively. They were conducted in nine countries and two territories from different regions of Asia, namely mainland China [25,26,27,28,29,30,31,32,33,34,35,36], Hong Kong–China [37,38], Taiwan–China [39], Korea [40], Turkey [41,42,43], Lebanon [44,45], Israel [46,47], Iran [48], India [49], Malaysia [50], and Thailand [51]. The majority of the studies (75%) took place in upper-middle income countries [25,26,27,28,29,30,31,32,33,34,35,36,41,42,43,44,45,46,49,51,52], followed by high-income countries (21.4%) [37,38,39,40,46,47] and low-middle income countries (3.6%) [49]. Most of these studies (85.7%) were carried out in urban areas or large cities [25,26,27,28,29,31,32,33,34,35,36,37,38,40,41,42,43,44,45,47,48,49,50,51]. The rest (14.3%) were carried out in rural areas [30,39,45,51]. Characteristics of included studies are summarised in Table 1.

Among CRCTs and RCTs, nine studies were categorised as high risk of bias mostly due to the lack of information on controlling possible bias for either the outcome measurement or from non-adherence, and selective reporting of findings [25,27,30,31,32,41,42,43,44,45,52]. The rest were studies with some concerns [28,34,37,39,48] or low risk of bias [29,33,36]. For the quasi-experiments, seven out of nine studies were rated as serious risk of bias [35,40,46,47,48,50,51] that was mostly due to insufficient control of possible bias from either possible confounders or dropouts, and/or selective reporting of findings. Other studies were rated as moderate risk of bias [38] or low risk of bias [26].

### 3.3. Characteristics of Interventions

Although the double burden of malnutrition has been the main problem in Asia for decades, almost all interventions (27 out of 28 studies) aimed to address childhood overweight and obesity. Only one study was conducted to tackle undernutrition [45]. Among the childhood overweight and obesity studies, eight studies were overweight/obesity reduction (included only overweight/obese children) [26,30,32,37,40,48,50], while the others were prevention studies (included children with all nutritional status). More information is shown in Table S1.

Six studies were carried out primarily by researchers in the health sector [25,27,28,29,34,35], while most studies were conducted by academic institutions. Five interventions co-developed relevant curriculums or programmes in collaboration with government agencies such as the Ministry of Education [31,44,47], educational and health authorities [42], and the local council [46] to gain cooperation from schools and other local stakeholders. Six interventions were the health sectors’ initiatives [25,27,28,29,34,35], while the rest did not seek government support to work with the schools. Detailed information is described in Table S2.

To implement the interventions, five studies were conducted entirely by investigators [32,38,39,40,43] and 23 studies engaged the schools and local actors. For the latter, teachers were engaged to provide nutrition education (NE) [28,29,45], physical activity promotion, such as physical education (PE), exercise prescriptions or/and enabling environment for active lifestyle [30,34,35,47], both NE and physical activity promotion [25,27,31,36,42,44,46,48,49], and to take students’ anthropometric measurement [51]. Kitchen or canteen staff were asked to provide healthier food [27,28,31,33,36,42,44,45,48,49]. Parents were trained to encourage a healthy diet and/or active lifestyle in children [25,26,27,28,29,31,37,41,42,44,47,48,49,51] (see Table S2).

The majority of studies (20 studies, 71.4%) implemented multiple-component interventions [25,26,27,28,29,30,31,32,33,36,37,40,42,45,46,47,48,49,50,51]. Among these studies, eight studies implemented nutrition education and extra exercise sessions [25,26,28,29,30,37,40,47]. Nine studies implemented nutrition education, extra exercise sessions, and additional components, such as healthier school food or lunch boxes, enabling school environments for an active lifestyle, psychological intervention, and individual consultations [31,32,33,36,42,44,46,48,49]. Two studies provided nutrition education and healthy meals including whole grains [50] and healthy snacks [45]. One study provided nutrition education and participatory eating events/campaigns [27]. Among the single-component interventions, five studies provided nutrition education [39,41,43,44,52] and three studies implemented extra exercise sessions [33,34,37] (see Table S2).

The sample sizes ranged from 32 to 8853 children. Among these, 21.4% were small studies (<100 students), 42.9% were medium studies (100–1000 students) and 32.7% were large studies (>1000 students). The duration of the interventions ranged from 8 weeks to five school years. Most interventions conducted 1-school-year programmes [25,26,27,28,32,33,34,36,41,45,46], the others conducted 8-week to 8-month programmes [30,35,37,39,40,42,43,44,49,51,52], 2-year programmes [31,44,47], 3-year programmes [29,45], and a 5-year programme [48] (see Table 1).

### 3.4. Impacts of Interventions

Most studies reported either BMI or BAZ. Of the 28 studies, 24 studies were eligible for the meta-analysis. Four studies were excluded because they did not report usable forms of outcomes, e.g., no information of standard deviation (SD), standard error (SE), or 95% confidence interval (CI) [35,41,46] or only addressed undernutrition which did not align with other studies [45]. Therefore, the overall effects described in this study are the effectiveness of the school nutrition interventions in reducing BMI or BAZ.

### 3.5. BMI

Fifteen studies (27 trials) reported BMI as an outcome. The pooled effect of the interventions was a reduced school-aged children’s BMI by −0.36 kg/m^2^ (95% CI: −0.46, −0.25).

**Figure 2:**
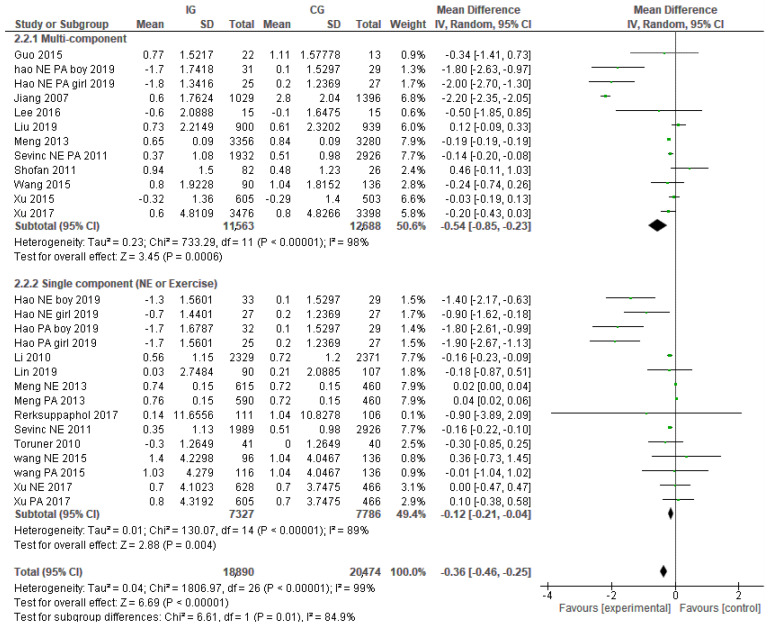
Overall effects on BMI and the difference between multi-component interventions and single-component interventions.

**Figure 3:**
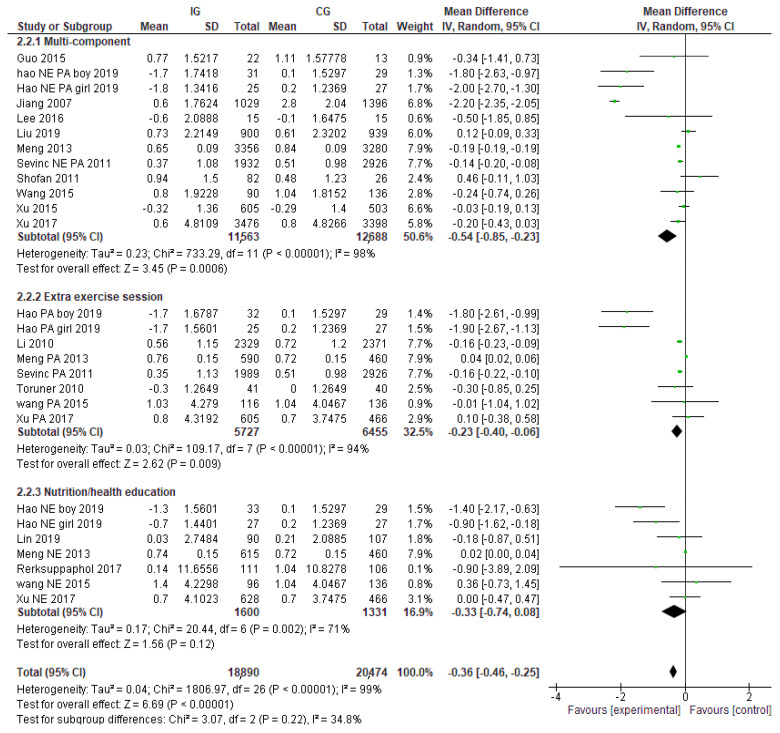
Pooled effects on BMI and the differences between multi-component interventions, nutrition education, and extra exercise prescription.

Subgroup analyses identified variation in effectiveness, depending on the type of intervention. The pooled effect size of multi-component interventions was higher than single-component interventions with BMI reductions of −0.54 (95% CI: −0.85, −0.23) kg/m^2^ and −0.12 kg/m^2^ (95% CI: −0.21, −0.04) respectively, (Figure 2). The confidence intervals of the two pooled effects do not overlap. Among the single-component interventions, extra exercise sessions significantly changed BMI with pooled effects of −0.23 kg/m^2^ (95% CI: −0.40, −0.06), while nutrition education did not show a significant change (−0.33 kg/m^2^ (95% CI: −0.74, 0.08), (See Figure 3).

The results of subgroup analyses for treatment and prevention interventions, showed that the pooled effect size of interventions aiming to reduce overweight/obesity was higher than interventions aiming to prevent it with the reductions of −0.94 kg/m^2^ (95% CI: −1.41, −0.47) and −0.23 kg/m^2^ (95% CI: −0.35, −0.12), respectively (see Figure S1). Five multi-component interventions were found in 12 overweight/obesity treatment interventions (42%), and six were found in 15 overweight/obesity prevention interventions (40%).

There was no significant difference in BMI reduction between interventions with and without parents’ participation with BMI reductions of −0.24 kg/m^2^ (−0.62, 0.14) and −0.29 kg/m^2^ (−0.41, −0.16), respectively (see Figure S2). The subgroup without parents’ participation contained a lower percentage of multi-component interventions (5 out of 15 trials, 33%) compared to the group with parents’ participation (6 out of 12 trials, 50%). The subgroup without parents’ participation and the group with parents’ participation contained overweight/obesity treatment equally at 5 out of 15 trials (33%) and 4 out of 12 trials (33%), respectively.

Subgroup analysis for interventions with and without healthy food provision showed that the interventions without healthy food provision group reduced BMI with a reduction of −0.77 kg/m^2^ (−1.34, −0.19), while the interventions with healthy food provision group did not show a significant reduction. Multi-component interventions were found more frequently in the interventions with healthy food provision than another group (9 out of 9 (100%) versus 12 out of 18 (67%), respectively). Twelve out of 18 interventions without healthy food provision (67%) were overweight/obesity treatment, while none was found in the subgroup of interventions with healthy food provision.

Subgroup analyses according to other characteristics of interest did not show significant differences between subgroups.

### 3.6. BAZ

The pooled effect of 15 studies (20 trials) reporting BAZ was a statistically significant reduction of −0.05 (95% CI: −0.08, −0.03).

**Figure 4:**
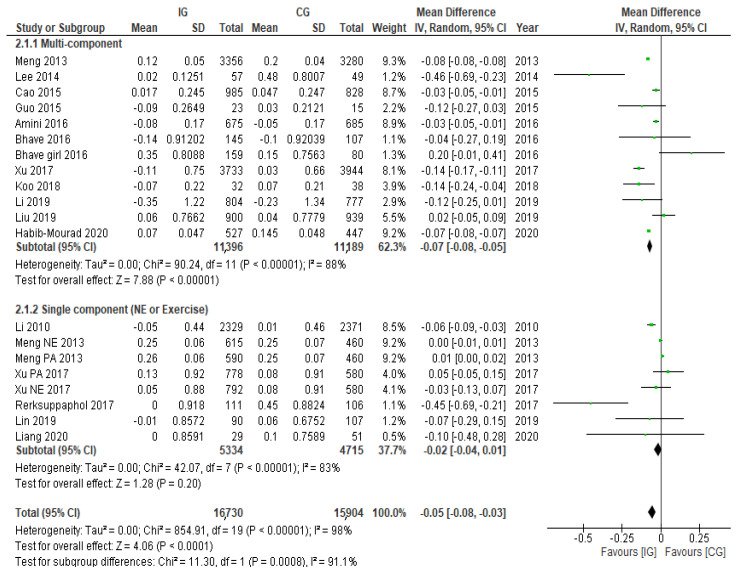
Pooled effects on BAZ and the difference between multi-component interventions and single component interventions.

**Figure 5:**
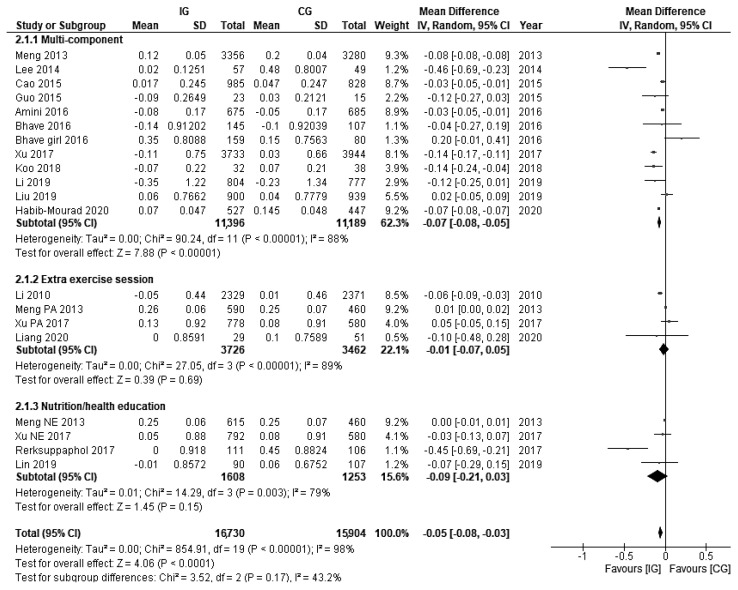
Pooled effects on BAZ and the differences between multi-component interventions, nutrition education and extra exercise prescription.

Categorised by the interventions’ component, the multi-component interventions significantly reduced BAZ by −0.07 (95% CI: −0.08, −0.05), while the pooled effect size of single-component interventions was not statistically significant (See Figure 4). No significant difference was found between nutrition education and extra exercise prescription (See Figure 5).

Similar to the change of BMI, overweight/obesity treatment interventions showed greater effect in reducing BAZ than overweight/obesity prevention interventions with the reductions of −0.15 (95% CI: −0.28, −0.02) and −0.05 (95% CI: −0.07, −0.02), respectively (see Figure S2). All overweight/obesity treatment interventions and six overweight/obesity prevention interventions of 16 studies (40%) were multi-component interventions.

The interventions with parents’ participation did not show an outstanding impact. These interventions provided a BAZ reduction of −0.05 (−0.09, −0.01), while the interventions without parents’ participation had a BAZ reduction of −0.06 (−0.10, −0.02) (see Figures S2 and S4). Also, there was no difference between the percentage of multi-component interventions among the with and without parents’ participation groups (6 out of 11 trials, 55% versus 5 out of 9 trials, 56%, respectively). Three out of 11 interventions without parents’ participation (21%) were overweight/obesity treatments, while only 1 out of 9 interventions with parents’ participation (11%) was an overweight/obesity treatment.

Subgroup analysis for interventions with and without healthy food provision showed slightly different BAZ reductions between the subgroups. The interventions without healthy food provision provided a BAZ reduction of −0.09 (−0.16, −0.03), while the interventions with healthy food provided a BAZ reduction of −0.04 (−0.07, −0.01). Nine out of 10 interventions with healthy food provision (90%) were multi-component interventions, while only 2 out of 10 interventions without healthy food provision (20%) were multi-component interventions. Overweight/obesity interventions equally belonged to interventions with and without healthy food provision subgroups.

Subgroup analyses according to other characteristics of interest did not show significant differences between subgroups.

### 3.7. Sensitivity Analysis and Publication Bias

The results of the sensitivity analysis indicated that there were no major changes of pooled effects after excluding studies with a high risk of bias or a quasi-experimental design, as shown in Figures S7 and S8. The BMI pooled effects sizes were −0.34 kg/m^2^ (95% CI: −0.49, −0.19, I^2^ = 99%) and −0.39 kg/m^2^ (95% CI: −0.50, −0.28, I^2^ = 99%), respectively, compared with the original −0.36 kg/m^2^ (95% CI: −0.46, −0.25, I^2^ = 99%).

**Figure 6:**
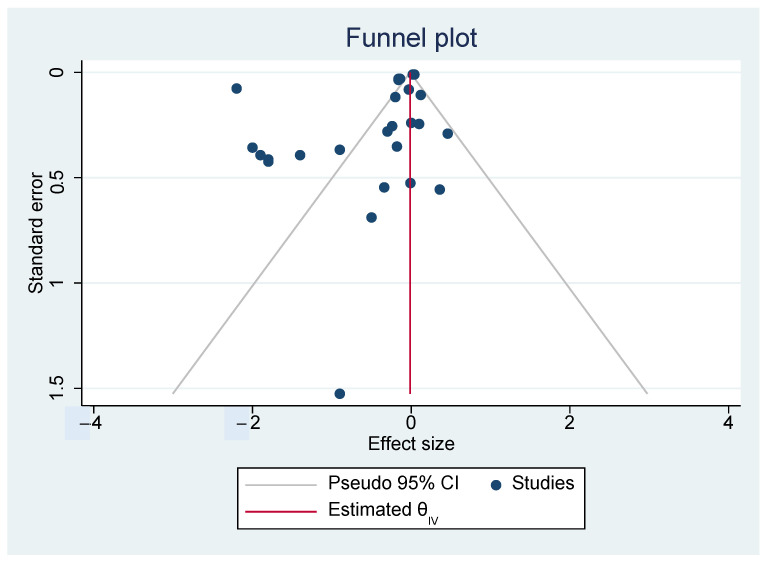
Funnel plot of effects.

Publication bias also was not detected. Even though the funnel plot of the studies’ effects on BMI was not perfectly symmetric (see Figure 6), the Egger’s regression test did not reject the null hypothesis (*p* = 0.2234).

## 4. Discussion

Our findings suggest that, in general, primary school nutrition interventions implemented in different Asian countries significantly reduced BMI and BAZ among school-aged children. However, the effectiveness varies with certain characteristics of the interventions. In terms of intervention components, multi-component interventions showed significant reductions for both BMI and BAZ, while single-component interventions showed a significant reduction only for BMI. In addition, multi-component interventions had a stronger effect than single-component interventions in reducing BMI. Among the single-component interventions, extra exercise sessions significantly reduced the BMI of the children, while nutrition education did not. In terms of intervention aim, overweight/obesity treatment provided stronger effects in reducing BMI and BAZ than overweight/obesity prevention interventions. Involving parents in the interventions did not significantly strengthen the effectiveness of the interventions. Interventions with school food improvement showed a smaller effect size than interventions without the component.

School nutrition interventions were effective in reducing BMI and BAZ among children of all ages in western/high-income countries and China [7,8,9,10]. This review adds to the current body of evidence that the interventions were effective in school-aged children in Asian countries as well. This accumulated evidence suggests that school nutrition interventions are promising measures in addressing childhood overweight/obesity across diverse contexts.

The results also suggested that multi-component interventions are more effective than single-component interventions, which are in line with the findings of meta-analyses from other contexts [8,9,10]. These findings emphasise the importance of a holistic approach in addressing childhood obesity. Among the single-component interventions, this review found that a significant reduction of BMI yielded from physical activity interventions but not nutrition education. A meta-analysis from China [10] also found a significant reduction of BMI from physical activity interventions, while the impacts of nutrition education were not reported. In terms of the educational strategies used in the trials, there was no major difference between nutrition education interventions and multi-component interventions. Most of the interventions were a classroom-based approach, and all interventions emphasised the importance of having a healthy diet, active lifestyle, and normal body weight, as well as provided guidance on body weight management through a variety of teaching materials. Thus, the different effects found from multi-component and nutrition education interventions may be due to the synergistic power of multiple components. The numbers of trials included in the physical activity and nutrition education subgroup analyses were not different (eight and seven trials, respectively), so the different effects between the interventions may not be due to the size of the analyses. Among the nutrition education interventions, only two trials showed significant BMI reductions. They were conducted in overweight/obese children who had never received nutrition education before [30], while most of the nutrition education interventions were conducted in general children who had received nutrition education. Therefore, the results indicated that additional nutrition education did not reduce mean BMI among children with mixed-nutritional statuses.

The stronger effect found on treatment than prevention of obesity and being overweight is in accordance with the findings from other meta-analyses in children of all ages [10,52]. This review indicated that the greater effects of overweight/obesity treatment were unlikely to be determined by the comprehensiveness of the interventions. This is because the numbers of multi-component interventions in treatment and prevention subgroups were found almost equally when analysing their impacts on BMI. Also, there were no clear distinctions between the treatment and prevention interventions in terms of the components included in those interventions. The information on the interventions’ implementation (e.g., fidelity, intensity, and adherence) was not clearly described in most studies, so it is challenging to examine the role of intervention implementation on the different effects. Given the lack of information, we are unable to identify the factors contributing to the different effect sizes between overweight/obesity treatment and prevention interventions.

This review also found that parent involvement did not significantly increase the effectiveness of school nutrition interventions, which is not in line with the findings from other meta-analyses [7,8]. A review of European childhood weight control interventions reported that medium- and high-intensity parental involvement (parents are directly involved in multiple activities and behaviour change methods in multiple sessions) were frequently found in effective interventions, while low-intensity parental involvement (parents are directly involved in one session and indirectly approached in three months period) was frequently found in less effective interventions [53]. The parental involvement of the school nutrition interventions included in this review can be categorised as low according to the criteria described above [54], since parents were invited to parental meetings once or twice with or without learning materials and only one study provided individual consultation for parents who had overweight/obese children. Therefore, whether or not parents are involved may not be the only answer, and the intensity of parental involvement may also play an important role in determining the effectiveness of the interventions.

This review found that interventions with healthy food provision significantly decreased BMI and BAZ of the children. It was also reported elsewhere that a healthy school food environment was effective in reducing students’ BAZ [55]. In addition to that, surprising findings were found by our subgroup analysis that interventions with healthy food provision included in this review provided weaker effects compared to interventions without the component. Theoretically, a healthy school food environment has the potential to play an important role in addressing childhood obesity since it could influence students’ diet [56,57] and diet is a key factor determining obesity [58]. There may be more factors influencing the unexpected results of this review. Considering the implementation of healthy food provision included in this review, there were variations in terms of the criteria for healthy food, ranging from whole grains to reduction of high caloric food and provision of fruit and vegetables. Also, most studies asked school kitchen staff to provide healthier choices, but there is no information whether food available in the schools met the criteria or not and to what extent the food affected the energy intake of the children. The United Nations also recognised that the results of school food on childhood obesity were not consistent, which may be due to the variation in school food provision, especially nutritional quality of school food, across different contexts [59]. Therefore, the level of food healthiness and adherence to the healthy food criteria could be the mediators. In addition, most interventions without healthy food provision included in this review were overweight/obesity treatment or multi-component interventions, of which generally provided stronger effects than prevention or single-component interventions. The opposites were found for interventions with healthy food provision. The different prevalence of treatment or multi-component interventions among the subgroups could be another influencer contributing to the different effects.

### 4.1. Policy Implications

Across all types of intervention, multi-component school nutrition interventions are the best option that provide consistent and strongest impacts in addressing childhood overnutrition. Among single-component interventions, extra exercise sessions have the potential to be mildly effective as a standalone component, while nutrition education should be a supplementary component.

Although parental involvement has been widely recognised as a promising strategy, insufficient involvement may compromise the effectiveness. To gain benefit from implementing a healthy food environment, the criteria for healthy food should comply with school nutrition standards and practice guidelines.

### 4.2. Future Research

The way primary studies reported the outcomes is important. Incomplete or unclear information restricts the ability to use the evidence. A significant proportion of studies selectively reported only certain forms of outcomes that are not comparable to the majority of literature, causing those studies to be excluded from secondary analyses. Also, not many studies provided clear information on intervention implementation (e.g., fidelity, intensity, and compliance), especially components related to food and physical activity environment and parental support. The lack of information compromised the usefulness of these included studies. Therefore, future evaluative studies on school nutrition interventions should provide complete information on both intervention implementation and outcomes. Existing tools such as the Template for Intervention Description and Replication (TIDieR) checklist [60] could be used to guide in the intervention reports. In addition, standard tools for food classification, such as nutrient profiling or school food standards, may help improve the intervention assessment regarding the nutritional quality of food provided to children.

### 4.3. Limitations

An interpretation of the findings of this review may require careful consideration due to the following limitations. Firstly, this review is restricted to English articles published in peer-reviewed journals, so some evidence published only in Asian languages might have been excluded. Secondly, a high degree of heterogeneity was detected in the pooled effect analysis. Sensitivity tests showed that there are no concerns related to study quality and study design. The school nutrition interventions are complex with variations of actors, intervention intensity, and surrounding environments. The complexity of interventions may be related to the considerable degree of heterogeneity. Thirdly, identifying factors contributing to the effects are not feasible. This is because the number of primary studies was not large enough, and the interventions’ contents were not clearly described for all studies.

## 5. Conclusions

Primary school nutrition interventions implemented in Asia are effective in reducing BMI and BAZ among school-aged children. Multi-component interventions provided promising outcomes in reducing the children’s BMI and BAZ. Among single-component interventions, extra exercise has the potential to reduce BMI, but nutrition education did not lead to significant changes. Overweight/obesity reduction interventions are more effective than overweight/obesity prevention interventions potentially due to different levels of intensity. Parental involvement and a healthy food provision do not always boost the effectiveness of school nutrition interventions, especially when the implementation is not sufficient. Comprehensiveness and intensity are key factors that must be considered seriously when designing school nutrition interventions to maximise the interventions’ effects. Studies assessing the impacts of school nutrition interventions should report complete information related to the interventions and outcomes to ensure their maximum benefit.

## Figures and Tables

**Table 1:** Summary of the studies included in this review.

Category	Number (%)
**Study design**	
RCTs	3 (10.7)
Cluster RCTs	15 (53.6)
Quasi-experiments	10 (35.7)
**Intervention component**	
Nutrition education	5 (17.9)
Extra exercise	3 (10.7)
Multi-component *	20 (71.4)
**Intervention duration**	
<1 school year	11 (39.3)
≥1 school year	17 (60.7)
**Sample size**	
<100 students	6 (21.4)
≥100–1000 students	12 (42.9)
>1000 students	10 (32.7)
**Country income**	
High-income country	6 (21.4)
Low and middle-income country	22 (78.6)
**Urbanity**	
Urban	24 (85.7)
Rural	4 (14.3)

* Nutrition education, extra exercise, enabling school environments for food and physical activity, psychological intervention, and individual consultations.

## Data Availability

The data presented in this study are openly available in references number 25 to 52 and the Supplementary Materials.

## Supplementary Materials

The following are available online at https://www.mdpi.com/article/10.3390/nu14030589/s1, Figure S1: Pooled effects on BMI, and the difference between overweight/obesity reduction and overweight/obesity prevention interventions, Figure S2: Pooled effects on BMI, and the difference between interventions with parents’ involvement and without parents’ involvement, Figure S3: Pooled effects on BMI, and the difference between interventions with healthy food provision and without, Figure S4: Pooled effects on BAZ, and the difference between overweight/obesity reduction and overweight/obesity prevention interventions, Figure S5: Pooled effects on BAZ, and the difference between interventions with parents’ involvement and without parents’ involvement, Figure S6: Pooled effects on BAZ, and the difference between interventions with healthy food provision and without, Figure S7: Sensitivity analysis: removing high risk of bias studies, Figure S8: Sensitivity analysis: removing quasi-experimental studies, Table S1: Characteristics of studies included in this review, Table S2: Intervention characteristics.

## References

[1] WHO Fact Sheets—Malnutrition. https://www.who.int/news-room/fact-sheets/detail/malnutrition.

[2] Development Initiatives (2020). 2020 Global Nutrition Report: Action on Equity to End Malnutrition, Bristol. https://globalnutritionreport.org/reports/2020-global-nutrition-report/.

[3] Abarca-Gómez L., Abdeen Z.A., Hamid Z.A., Abu-Rmeileh N.M., Acosta-Cazares B., Acuin C., Adams R.J., Aekplakorn W., Afsana K., Aguilar-Salinas C.A. (2017). Worldwide trends in body-mass index, underweight, overweight, and obesity from 1975 to 2016: A pooled analysis of 2416 population-based measurement studies in 1289 million children, adolescents, and adults. Lancet.

[4] Peña M., Bacallao J. (2022). Malnutrition and poverty. Annu. Rev. Nutr..

[5] School Feeding|World Food Programme. https://www.wfp.org/school-meals.

[6] WHO (2014). Nutrition-Friendly Schools Initiative (NFSI).

[7] Katz D., O’Connell M., Njike V., Yeh M., Nawaz H. (2008). Strategies for the prevention and control of obesity in the school setting: Systematic review and meta-analysis. Int. J. Obes..

[8] Sobol-Goldberg S., Rabinowitz J., Gross R. (2013). School-based obesity prevention programs: A meta-analysis of randomized controlled trials. Obesity.

[9] Liu Z., Xu H.-M., Wen L.-M., Peng Y.-Z., Lin L.-Z., Zhou S., Li W.-H., Wang H.-J. (2019). A systematic review and meta-analysis of the overall effects of school-based obesity prevention interventions and effect differences by intervention components. Int. J. Behav. Nutr. Phys. Act..

[10] Feng L., Wei D.M., Lin S.T., Maddison R., Ni Mhurchu C., Jiang Y., Gao Y., Wang H.J. (2017). Systematic review and meta-analysis of school-based obesity interventions in mainland China. PLoS ONE.

[11] Adom T., De Villiers A., Puoane T., Kengne A.P. (2020). School-based interventions targeting nutrition and physical activity, and body weight status of African children: A systematic review. Nutrients.

[12] Kyere P., Veerman J.L., Lee P., Stewart D.E. (2020). Effectiveness of school-based nutrition interventions in sub-Saharan Africa: A systematic review. Public Health Nutr..

[13] Verstraeten R., Roberfroid M., Lachat C., Leroy J.L., Holdsworth M., Maes L., Kolsteren P.W. (2012). Effectiveness of preventive school-based obesity interventions in low- and middle-income countries: A systematic review. Am. J. Clin. Nutr..

[14] Eccles J.S. (1999). The development of children ages 6 to 14. Future Child..

[15] Vereecken C.A., Bobelijn K., Maes L. (2005). School food policy at primary and secondary schools in Belgium-Flanders: Does it influence young people’s food habits?. Eur. J. Clin. Nutr..

[16] Scholtens S., Middelbeek L., Rutz S.I., Buijs G., Bemelmans W.J. (2010). Differences in school environment, school policy and actions regarding overweight prevention between Dutch schools. A nationwide survey. BMC Public Health.

[17] UNESCO (2020). Global Education Monitoring Report 2020: Inclusion and Education: All Means All.

[18] Fink G., Rockers P.C. (2014). Childhood growth, schooling, and cognitive development: Further evidence from the Young Lives study. Am J Clin Nutr..

[19] Davies P. (2012). The State of Evidence-Based Policy Evaluation and its Role in Policy Formation. Natl. Inst. Econ. Rev..

[20] Moher D., Shamseer L., Clarke M., Ghersi D., Liberati A., Petticrew M., Shekelle P., Stewart L.A., PRISMA-P Group (2015). Preferred reporting items for systematic review and meta-analysis protocols (PRISMA-P) 2015 statement. Syst. Rev..

[21] Eldridge S., Campbell M., Campbell M., Drahota-Towns A., Giraudeau B., Higgins J., Reeves B., Siegfried N. (2016). Revised Cochrane Risk of Bias Tool for Randomized Trials (RoB 2.0): Additional Considerations for Cluster-Randomized Trials (Working Paper). https://sites.google.com/site/riskofbiastool/welcome/rob-2-0-tool.

[22] Sterne J.A.C., Hernán M.A., Reeves B.C., Savović J., Berkman N.D., Viswanathan M., Henry D., Altman D.G., Ansari M.T., Boutron I. (2016). ROBINS-I: A tool for assessing risk of bias in non-randomised studies of interventions. BMJ.

[23] Egger M., Smith G.D., Schneider M., Minder C. (1997). Bias in meta-analysis detected by a simple, graphical test. BMJ.

[24] Xu H., Li Y., Zhang Q., Hu X.L., Liu A., Du S., Li T., Guo H., Li Y., Xu G. (2017). Comprehensive school-based intervention to control overweight and obesity in China: A cluster randomized controlled trial. Asia Pac. J. Clin. Nutr..

[25] Wang J., Lau W.P., Wang H., Ma J. (2015). Evaluation of a comprehensive intervention with a behavioural modification strategy for childhood obesity prevention: A nonrandomized cluster controlled trial. BMC Public Health.

[26] Xu F., Ware R.S., Leslie E., Tse L.A., Wang Z., Li J., Wang Y. (2015). Effectiveness of a randomized controlled lifestyle intervention to prevent obesity among Chinese primary school students: Click-obesity study. PLoS ONE.

[27] Meng L., Xu H., Liu A., Van Raaij J., Bemelmans W., Hu X., Zhang Q., Du S., Fang H., Ma J. (2013). The costs and cost-effectiveness of a school-based comprehensive intervention study on childhood obesity in China. PLoS ONE.

[28] Jiang J., Xia X., Greiner T., Wu G., Lian G., Rosenqvist U. (2007). The effects of a 3-year obesity intervention in schoolchildren in Beijing. Child Care Health Dev..

[29] Hao M., Han W., Yamauchi T. (2019). Short-Term and Long-Term Effects of a Combined Intervention of Rope Skipping and Nutrition Education for Overweight Children in Northeast China. Asia-Pac. J. Public Health.

[30] Cao Z.J., Wang S.M., Chen Y. (2015). A randomized trial of multiple interventions for childhood obesity in china. Am. J. Prev. Med..

[31] Guo H., Zeng X., Zhuang Q., Zheng Y., Chen S. (2015). Intervention of childhood and adolescents obesity in Shantou city. Obes. Res. Clin. Pract..

[32] Li B., Pallan M., Liu W.J., Hemming K., Frew E., Lin R., Martin J., Zanganeh M., Hurley K., Cheng K.K. (2019). The CHIRPY DRAGON intervention in preventing obesity in Chinese primary-school-aged children: A cluster-randomised controlled trial. PLoS Med..

[33] Li Y.-P., Hu X.-Q., Schouten E.G., Liu A.-L., DU S.-M., Li L.-Z., Cui Z.-H., Wang D., Kok F.J., Hu F.B. (2010). Report on Childhood Obesity in China (8): Effects and Sustainability of Physical Activity Intervention on Body Composition of Chinese Youth. Biomed. Environ. Sci..

[34] Liu A., Hu X., Ma G., Cui Z., Pan Y., Chang S., Zhao W.C.C. (2008). Evaluation of a classroom-based physical activity promoting programme. Obeity Rev..

[35] Liu Z., Li Q., Maddison R., Ni Mhurchu C., Jiang Y., Wei D.-M., Cheng L., Cheng Y., Wang D., Wang H.-J. (2019). A School-Based Comprehensive Intervention for Childhood Obesity in China: A Cluster Randomized Controlled Trial. Child Obes..

[36] Lee A., Ho M., Keung V.M.W., Kwong A.C.M. (2014). Childhood obesity management shifting from health care system to school system: Intervention study of school-based weight management programme. BMC Public Health.

[37] Liang Y., Lau P.W.C., Jiang Y., Maddison R. (2020). Getting Active with Active Video Games: A Quasi-Experimental Study. Int. J. Environ. Res. Public Health.

[38] Lin Y.-C., Chen H.-J., Wang Y., Min J., Wu H.-C., Carvajal N.A., Yang H.-Y. (2019). NASA Mission X Program for Healthy Eating and Active Living among Taiwanese Elementary School Students. J. Pediatr. Nurs..

[39] Lee G., Choi Y. (2016). Effects of an obesity management mentoring program for Korean children. Appl. Nurs. Res..

[40] Akdemir M., Donmez L., Polat H.H. (2007). The Effect of Nutritional and Physical Activity Interventions on Nutritional Status and Obesity in Primary School Children: A Cluster Randomized Controlled Study. Kuwait Med. J..

[41] Sevinç Ö., Bozkurt A.I., Gündoğdu M., Aslan Ü.B., Ağbuğa B., Aslan Ş., Dikbaş E., Gökçe Z. (2011). Evaluation of the effectiveness of an intervention program on preventing childhood obesity in Denizli, Turkey. Turk. J. Med. Sci..

[42] Toruner E.K., Ayaz S., Altay N., Citak E.A., Sahin S. (2015). Efficacy of a school-based healthy life program in Turkey. Child. Health Care.

[43] Toruner E.K., Savaser S. (2010). A controlled evaluation of a school-based obesity prevention in Turkish school children. J. Sch. Nurs..

[44] Habib-Mourad C., Ghandour L.A., Maliha C., Dagher M., Kharroubi S., Hwalla N. (2020). Impact of a three-year obesity prevention study on healthy behaviors and bmi among lebanese schoolchildren: Findings from ajyal salima program. Nutrients.

[45] El Harake M.D., Kharroubi S., Hamadeh S.K., Jomaa L. (2018). Impact of a pilot school-based nutrition intervention on dietary knowledge, attitudes, behavior and nutritional status of syrian refugee children in the bekaa, lebanon. Nutrients.

[46] Aperman-Itzhak T., Yom-Tov A., Vered Z., Waysberg R., Livne I., Eilat-Adar S. (2018). School-Based Intervention to Promote a Healthy Lifestyle and Obesity Prevention Among Fifth- and Sixth-Grade Children. Am. J. Health Educ..

[47] Shofan Y., Kedar O., Branski D., Berry E., Wilschanski M. (2011). A school-based program of physical activity may prevent obesity. Eur. J. Clin. Nutr..

[48] Amini M., Djazayery A., Majdzadeh R., Taghdisi M.-H., Sadrzadeh-Yeganeh H., Abdollahi Z., Hosseinpour-Niazi N., Chamari M., Nourmohammadi M. (2016). A School-Based Intervention to Reduce Excess Weight in Overweight and Obese Primary School Students. Biol. Res. Nurs..

[49] Bhave S., Pandit A., Yeravdekar R., Madkaikar V., Chinchwade T., Shaikh N., Shaikh T., Naik S., Marley-Zagar E., Fall C.H. (2016). Effectiveness of a 5-year school-based intervention programme to reduce adiposity and improve fitness and lifestyle in Indian children; the SYM-KEM study. Arch. Dis. Child..

[50] Koo H.C., Poh B.K., Talib R.A. (2018). The GReat-child^TM^ trial: A quasi-experimental intervention on whole grains with healthy balanced diet to manage childhood obesity in Kuala Lumpur, Malaysia. Nutrients.

[51] Rerksuppaphol L., Rerksuppaphol S. (2017). Internet based obesity prevention program for Thai school children-a randomized control trial. J. Clin. Diagn. Res..

[52] Lavelle H.V., MacKay D.F., Pell J.P. (2012). Systematic review and meta-analysis of school-based interventions to reduce body mass index. J. Public Health.

[53] Van Der Kruk J.J., Kortekaas F., Lucas C., Jager-Wittenaar H. (2013). Obesity: A systematic review on parental involvement in long-term European childhood weight control interventions with a nutritional focus. Obes. Rev..

[54] Donkersgoed H., Lanting L. (2010). Bereik van Doelgroepen Range of groupsBereik van Doelgroepen Range of Groups.

[55] Pineda E., Bascunan J., Sassi F. (2021). Improving the school food environment for the prevention of childhood obesity: What works and what doesn’t. Obes. Rev..

[56] FAO (2021). Healthy Food Environment and School Food|School Food and Nutrition|Food and Agriculture Organization of the United Nations. https://www.fao.org/school-food/areas-work/food-environment/en/.

[57] (2009). WHO Library Cataloguing-in-Publication Data Interventions on Diet and Physical Activity: What Works: Summary Report. www.blossoming.it.

[58] WHO, FAO (2003). Diet, Nutrition and the Prevention of Chronic Diseases: Recommendations for Preventing Excess Weight Gains and Obesity.

[59] FAO (2019). Nutrition Guidelines and Standards for School Meals: A Report from 33 Low and Middle-Income Countries.

[60] Hoffmann T.C., Glasziou P.P., Boutron I., Milne R., Perera R., Moher D., Altman D.G., Barbour V., Macdonald H., Johnston M. (2014). Better reporting of interventions: Template for intervention description and replication (TIDieR) checklist and guide. BMJ.

