# Supplementary material for: Impacts of School Nutrition Interventions on the Nutritional Status of School-Aged Children in Asia: A Systematic Review and Meta-Analysis
Source: Nutrients. 2022 Jan 28;14(3):589. doi: 10.3390/nu14030589 (PMC8839996; doi:10.3390/nu14030589)
Supplement: Supplementary file 1 [file nutrients-14-00589-s001.zip › nutrients-1536587-supplementary.pdf]

Supplements

Study or Subgroup	IG Mean	IG SD	IG Total	CG Mean	CG SD	CG Total	Weight	Mean Difference IV, Random, 95% CI
**2.2.1 Obesity treatment**								
Guo 2015	0.77	1.5217	22	1.11	1.57778	13	0.9%	-0.34 [-1.41, 0.73]
Hao NE boy 2019	-1.3	1.5601	33	0.1	1.5297	29	1.5%	-1.40 [-2.17, -0.63]
Hao NE girl 2019	-0.7	1.4401	27	0.2	1.2369	27	1.7%	-0.90 [-1.62, -0.18]
hao NE PA boy 2019	-1.7	1.7418	31	0.1	1.5297	29	1.3%	-1.80 [-2.63, -0.97]
Hao NE PA girl 2019	-1.8	1.3416	25	0.2	1.2369	27	1.7%	-2.00 [-2.70, -1.30]
Hao PA boy 2019	-1.7	1.6787	32	0.1	1.5297	29	1.4%	-1.80 [-2.61, -0.99]
Hao PA girl 2019	-1.7	1.5601	25	0.2	1.2369	27	1.5%	-1.90 [-2.67, -1.13]
Lee 2016	-0.6	2.0888	15	-0.1	1.6475	15	0.6%	-0.50 [-1.85, 0.85]
Toruner 2010	-0.3	1.2649	41	0	1.2649	40	2.5%	-0.30 [-0.85, 0.25]
Wang 2015	0.8	1.9228	90	1.04	1.8152	136	2.8%	-0.24 [-0.74, 0.26]
wang NE 2015	1.4	4.2298	96	1.04	4.0467	136	0.8%	0.36 [-0.73, 1.45]
wang PA 2015	1.03	4.279	116	1.04	4.0467	136	0.9%	-0.01 [-1.04, 1.02]
**Subtotal (95% CI)**			**553**			**644**	**17.6%**	**-0.94 [-1.41, -0.47]**
Heterogeneity: Tau² = 0.51; Chi² = 47.49, df = 11 (P < 0.00001); I² = 77%								
Test for overall effect: Z = 3.89 (P < 0.0001)								
**2.2.2 Obesity prevention**								
Jiang 2007	0.6	1.7624	1029	2.8	2.04	1396	6.8%	-2.20 [-2.35, -2.05]
Li 2010	0.56	1.15	2329	0.72	1.2	2371	7.7%	-0.16 [-0.23, -0.09]
Lin 2019	0.03	2.7484	90	0.21	2.0885	107	1.8%	-0.18 [-0.87, 0.51]
Liu 2019	0.73	2.2149	900	0.61	2.3202	939	6.0%	0.12 [-0.09, 0.33]
Meng 2013	0.65	0.09	3356	0.84	0.09	3280	7.9%	-0.19 [-0.19, -0.19]
Meng NE 2013	0.74	0.15	615	0.72	0.15	460	7.9%	0.02 [0.00, 0.04]
Meng PA 2013	0.76	0.15	590	0.72	0.15	460	7.9%	0.04 [0.02, 0.06]
Rerksuppaphol 2017	0.14	11.6556	111	1.04	10.8278	106	0.1%	-0.90 [-3.89, 2.09]
Sevinc NE 2011	0.35	1.13	1989	0.51	0.98	2926	7.7%	-0.16 [-0.22, -0.10]
Sevinc NE PA 2011	0.37	1.08	1932	0.51	0.98	2926	7.7%	-0.14 [-0.20, -0.08]
Shofan 2011	0.94	1.5	82	0.48	1.23	26	2.4%	0.46 [-0.11, 1.03]
Xu 2015	-0.32	1.36	605	-0.29	1.4	503	6.6%	-0.03 [-0.19, 0.13]
Xu 2017	0.6	4.8109	3476	0.8	4.8266	3398	5.8%	-0.20 [-0.43, 0.03]
Xu NE 2017	0.7	4.1023	628	0.7	3.7475	466	3.1%	0.00 [-0.47, 0.47]
Xu PA 2017	0.8	4.3192	605	0.7	3.7475	466	2.9%	0.10 [-0.38, 0.58]
**Subtotal (95% CI)**			**18337**			**19830**	**82.4%**	**-0.23 [-0.35, -0.12]**
Heterogeneity: Tau² = 0.03; Chi² = 1715.33, df = 14 (P < 0.00001); I² = 99%								
Test for overall effect: Z = 4.03 (P < 0.0001)								
**Total (95% CI)**			**18890**			**20474**	**100.0%**	**-0.36 [-0.46, -0.25]**
Heterogeneity: Tau² = 0.04; Chi² = 1806.97, df = 26 (P < 0.00001); I² = 99%								
Test for overall effect: Z = 6.69 (P < 0.00001)								
Test for subgroup differences: Chi² = 8.09, df = 1 (P = 0.004), I² = 87.6%								

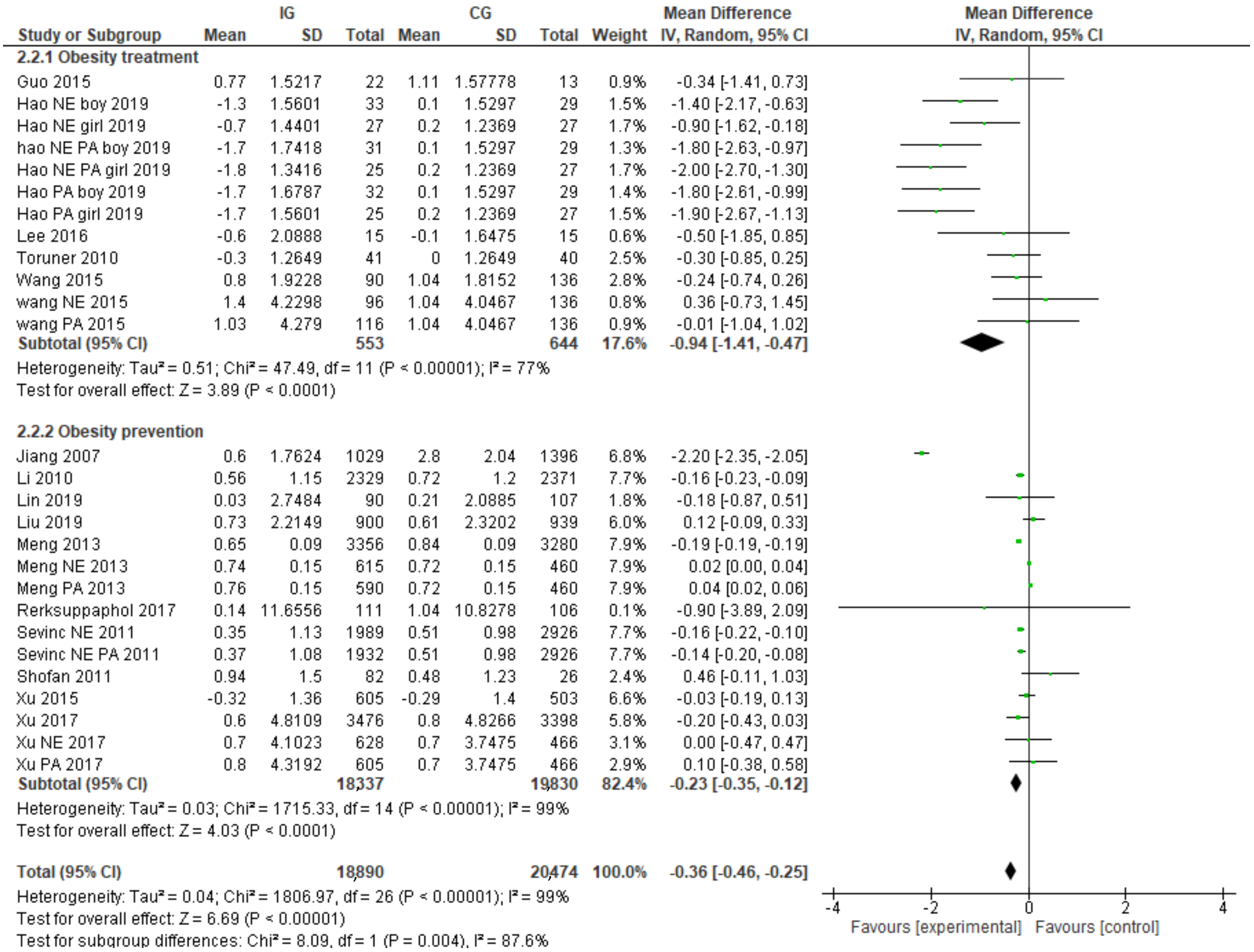

Figure S1. Pooled effects on BMI, and the difference between overweight/obesity reduction and overweight/obesity prevention interventions

Study or Subgroup	IG Mean	IG SD	IG Total	CG Mean	CG SD	CG Total	Weight	Mean Difference IV, Random, 95% CI
**2.2.2 Did not involve parents**								
Guo 2015	0.77	1.5217	22	1.11	1.57778	13	0.9%	-0.34 [-1.41, 0.73]
Hao NE boy 2019	-1.3	1.5601	33	0.1	1.5297	29	1.5%	-1.40 [-2.17, -0.63]
Hao NE girl 2019	-0.7	1.4401	27	0.2	1.2369	27	1.7%	-0.90 [-1.62, -0.18]
hao NE PA boy 2019	-1.7	1.7418	31	0.1	1.5297	29	1.3%	-1.80 [-2.63, -0.97]
Hao NE PA girl 2019	-1.8	1.3416	25	0.2	1.2369	27	1.7%	-2.00 [-2.70, -1.30]
Hao PA boy 2019	-1.7	1.6787	32	0.1	1.5297	29	1.4%	-1.80 [-2.61, -0.99]
Hao PA girl 2019	-1.7	1.5601	25	0.2	1.2369	27	1.5%	-1.90 [-2.67, -1.13]
Lee 2016	-0.6	2.0888	15	-0.1	1.6475	15	0.6%	-0.50 [-1.85, 0.85]
Li 2010	0.56	1.15	2329	0.72	1.2	2371	7.7%	-0.16 [-0.23, -0.09]
Lin 2019	0.03	2.7484	90	0.21	2.0885	107	1.8%	-0.18 [-0.87, 0.51]
Liu 2019	0.73	2.2149	900	0.61	2.3202	939	6.0%	0.12 [-0.09, 0.33]
Meng 2013	0.65	0.09	3356	0.84	0.09	3280	7.9%	-0.19 [-0.19, -0.19]
Meng NE 2013	0.74	0.15	615	0.72	0.15	460	7.9%	0.02 [0.00, 0.04]
Meng PA 2013	0.76	0.15	590	0.72	0.15	460	7.9%	0.04 [0.02, 0.06]
Rerksuppaphol 2017	0.14	11.6556	111	1.04	10.8278	106	0.1%	-0.90 [-3.89, 2.09]
**Subtotal (95% CI)**			**8201**			**7919**	**49.9%**	**-0.29 [-0.41, -0.16]**
Heterogeneity: Tau² = 0.02; Chi² = 1107.64, df = 14 (P < 0.00001); I² = 99%								
Test for overall effect: Z = 4.38 (P < 0.0001)								
**2.2.3 Involved parents**								
Jiang 2007	0.6	1.7624	1029	2.8	2.04	1396	6.8%	-2.20 [-2.35, -2.05]
Sevinc NE PA 2011	0.37	1.08	1932	0.51	0.98	2926	7.7%	-0.14 [-0.20, -0.08]
Sevinc PA 2011	0.35	1.13	1989	0.51	0.98	2926	7.7%	-0.16 [-0.22, -0.10]
Shofan 2011	0.94	1.5	82	0.48	1.23	26	2.4%	0.46 [-0.11, 1.03]
Toruner 2010	-0.3	1.2649	41	0	1.2649	40	2.5%	-0.30 [-0.85, 0.25]
Wang 2015	0.8	1.9228	90	1.04	1.8152	136	2.8%	-0.24 [-0.74, 0.26]
wang NE 2015	1.4	4.2298	96	1.04	4.0467	136	0.8%	0.36 [-0.73, 1.45]
wang PA 2015	1.03	4.279	116	1.04	4.0467	136	0.9%	-0.01 [-1.04, 1.02]
Xu 2015	-0.32	1.36	605	-0.29	1.4	503	6.6%	-0.03 [-0.19, 0.13]
Xu 2017	0.6	4.8109	3476	0.8	4.8266	3398	5.8%	-0.20 [-0.43, 0.03]
Xu NE 2017	0.7	4.1023	628	0.7	3.7475	466	3.1%	0.00 [-0.47, 0.47]
Xu PA 2017	0.8	4.3192	605	0.7	3.7475	466	2.9%	0.10 [-0.38, 0.58]
**Subtotal (95% CI)**			**10689**			**12555**	**50.1%**	**-0.24 [-0.62, 0.14]**
Heterogeneity: Tau² = 0.39; Chi² = 671.40, df = 11 (P < 0.00001); I² = 98%								
Test for overall effect: Z = 1.24 (P = 0.22)								
**Total (95% CI)**			**18890**			**20474**	**100.0%**	**-0.36 [-0.46, -0.25]**
Heterogeneity: Tau² = 0.04; Chi² = 1806.97, df = 26 (P < 0.00001); I² = 99%								
Test for overall effect: Z = 6.69 (P < 0.00001)								
Test for subgroup differences: Chi² = 0.05, df = 1 (P = 0.83), I² = 0%								

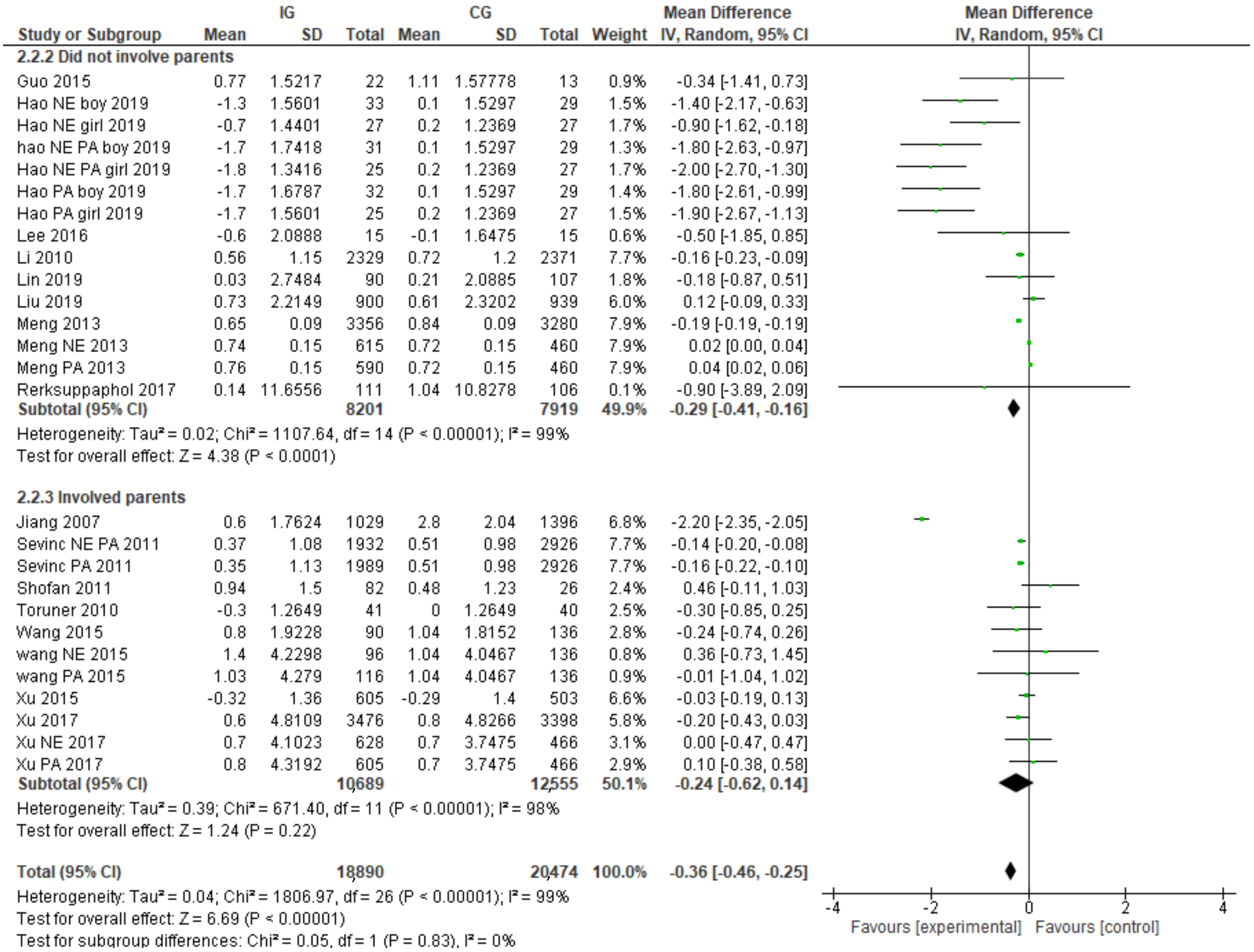

Figure S2. Pooled effects on BMI, and the difference between interventions with parents' involvement and without parents' involvement

Study or Subgroup	IG Mean	IG SD	IG Total	CG Mean	CG SD	CG Total	Weight	Mean Difference IV, Random, 95% CI
**2.2.2 With healthy food provision**								
Liu 2019	0.73	2.2149	900	0.61	2.3202	939	6.0%	0.12 [-0.09, 0.33]
Meng 2013	0.65	0.09	3356	0.84	0.09	3280	7.9%	-0.19 [-0.19, -0.19]
Meng NE 2013	0.74	0.15	615	0.72	0.15	460	7.9%	0.02 [0.00, 0.04]
Meng PA 2013	0.76	0.15	590	0.72	0.15	460	7.9%	0.04 [0.02, 0.06]
Sevinc NE 2011	0.35	1.13	1989	0.51	0.98	2926	7.7%	-0.16 [-0.22, -0.10]
Sevinc NE PA 2011	0.37	1.08	1932	0.51	0.98	2926	7.7%	-0.14 [-0.20, -0.08]
Xu 2015	-0.32	1.36	605	-0.29	1.4	503	6.6%	-0.03 [-0.19, 0.13]
Xu 2017	0.6	4.8109	3476	0.8	4.8266	3398	5.8%	-0.20 [-0.43, 0.03]
Xu NE 2017	0.7	4.1023	628	0.7	3.7475	466	3.1%	0.00 [-0.47, 0.47]
**Subtotal (95% CI)**			**14091**			**15358**	**60.7%**	**-0.07 [-0.17, 0.04]**
Heterogeneity: Tau² = 0.02; Chi² = 1021.03, df = 8 (P < 0.00001); I² = 99%								
Test for overall effect: Z = 1.23 (P = 0.22)								
**2.2.3 Without**								
Guo 2015	0.77	1.5217	22	1.11	1.57778	13	0.9%	-0.34 [-1.41, 0.73]
Hao NE boy 2019	-1.3	1.5601	33	0.1	1.5297	29	1.5%	-1.40 [-2.17, -0.63]
Hao NE girl 2019	-0.7	1.4401	27	0.2	1.2369	27	1.7%	-0.90 [-1.62, -0.18]
hao NE PA boy 2019	-1.7	1.7418	31	0.1	1.5297	29	1.3%	-1.80 [-2.63, -0.97]
Hao NE PA girl 2019	-1.8	1.3416	25	0.2	1.2369	27	1.7%	-2.00 [-2.70, -1.30]
Hao PA boy 2019	-1.7	1.6787	32	0.1	1.5297	29	1.4%	-1.80 [-2.61, -0.99]
Hao PA girl 2019	-1.7	1.5601	25	0.2	1.2369	27	1.5%	-1.90 [-2.67, -1.13]
Jiang 2007	0.6	1.7624	1029	2.8	2.04	1396	6.8%	-2.20 [-2.35, -2.05]
Lee 2016	-0.6	2.0888	15	-0.1	1.6475	15	0.6%	-0.50 [-1.85, 0.85]
Li 2010	0.56	1.15	2329	0.72	1.2	2371	7.7%	-0.16 [-0.23, -0.09]
Lin 2019	0.03	2.7484	90	0.21	2.0885	107	1.8%	-0.18 [-0.87, 0.51]
Rerksuppaphol 2017	0.14	11.6556	111	1.04	10.8278	106	0.1%	-0.90 [-3.89, 2.09]
Shofan 2011	0.94	1.5	82	0.48	1.23	26	2.4%	0.46 [-0.11, 1.03]
Toruner 2010	-0.3	1.2649	41	0	1.2649	40	2.5%	-0.30 [-0.85, 0.25]
Wang 2015	0.8	1.9228	90	1.04	1.8152	136	2.8%	-0.24 [-0.74, 0.26]
wang NE 2015	1.4	4.2298	96	1.04	4.0467	136	0.8%	0.36 [-0.73, 1.45]
wang PA 2015	1.03	4.279	116	1.04	4.0467	136	0.9%	-0.01 [-1.04, 1.02]
Xu PA 2017	0.8	4.3192	605	0.7	3.7475	466	2.9%	0.10 [-0.38, 0.58]
**Subtotal (95% CI)**			**4799**			**5116**	**39.3%**	**-0.77 [-1.34, -0.19]**
Heterogeneity: Tau² = 1.36; Chi² = 658.65, df = 17 (P < 0.00001); I² = 97%								
Test for overall effect: Z = 2.59 (P = 0.009)								
**Total (95% CI)**			**18890**			**20474**	**100.0%**	**-0.36 [-0.46, -0.25]**
Heterogeneity: Tau² = 0.04; Chi² = 1806.97, df = 26 (P < 0.00001); I² = 99%								
Test for overall effect: Z = 6.69 (P < 0.00001)								
Test for subgroup differences: Chi² = 5.42, df = 1 (P = 0.02), I² = 81.5%								

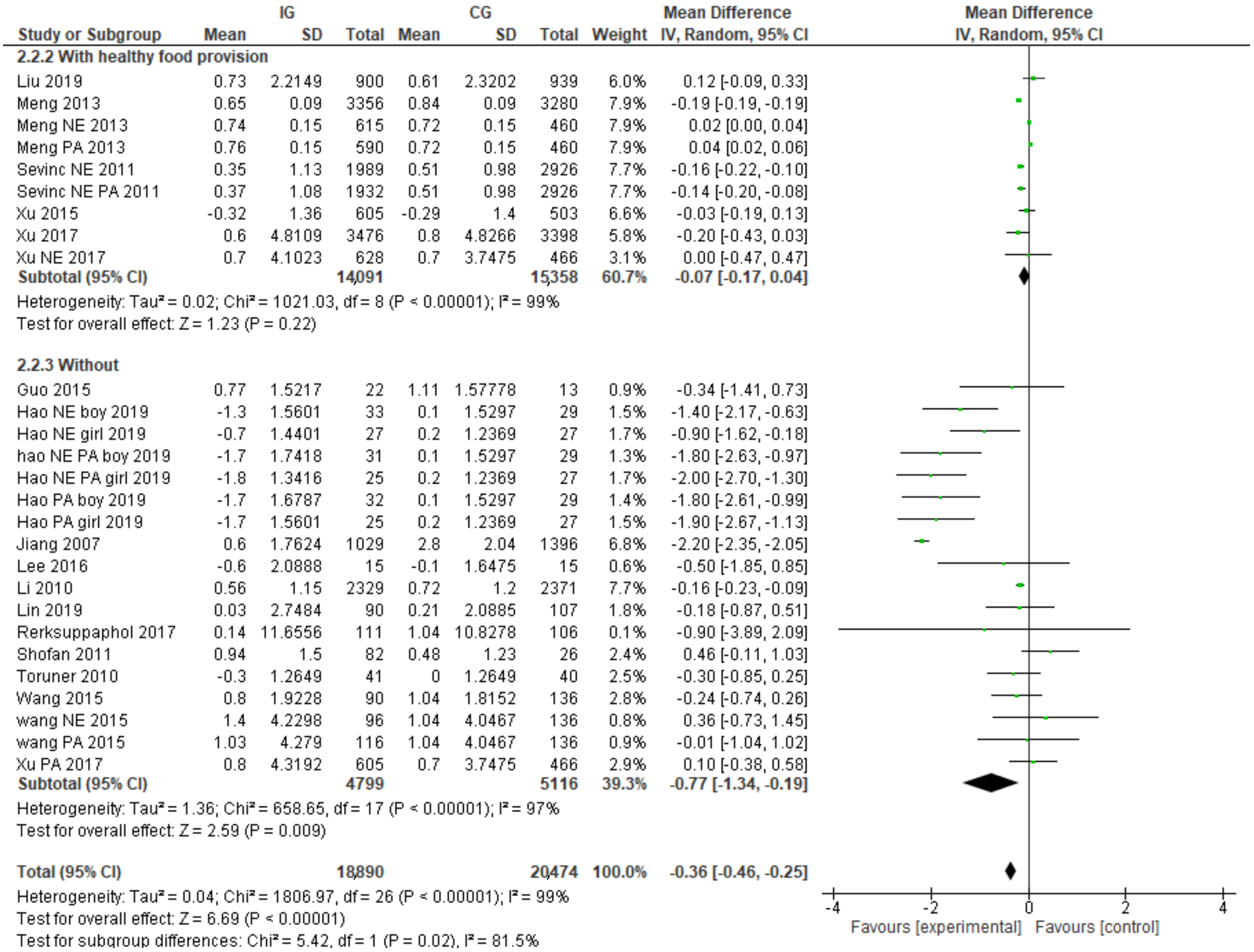

Figure S3. Pooled effects on BMI, and the difference between interventions with healthy food provision and without

Study or Subgroup	IG Mean	IG SD	IG Total	CG Mean	CG SD	CG Total	Weight	Mean Difference IV, Random, 95% CI	Year
**2.1.1 Obesity treatment**									
Lee 2014	0.02	0.1251	57	0.48	0.8007	49	1.2%	-0.46 [-0.69, -0.23]	2014
Guo 2015	-0.09	0.2649	23	0.03	0.2121	15	2.2%	-0.12 [-0.27, 0.03]	2015
Amini 2016	-0.08	0.17	675	-0.05	0.17	685	8.9%	-0.03 [-0.05, -0.01]	2016
Koo 2018	-0.07	0.22	32	0.07	0.21	38	3.9%	-0.14 [-0.24, -0.04]	2018
**Subtotal (95% CI)**			**787**			**787**	**16.1%**	**-0.15 [-0.28, -0.02]**	
Heterogeneity: Tau² = 0.01; Chi² = 19.08, df = 3 (P = 0.0003); I² = 84%									
Test for overall effect: Z = 2.28 (P = 0.02)									
**2.1.2 Obesity prevention**									
Li 2010	-0.05	0.44	2329	0.01	0.46	2371	8.5%	-0.06 [-0.09, -0.03]	2010
Meng 2013	0.12	0.05	3356	0.2	0.04	3280	9.3%	-0.08 [-0.08, -0.08]	2013
Meng NE 2013	0.25	0.06	615	0.25	0.07	460	9.2%	0.00 [-0.01, 0.01]	2013
Meng PA 2013	0.26	0.06	590	0.25	0.07	460	9.2%	0.01 [0.00, 0.02]	2013
Cao 2015	0.017	0.245	985	0.047	0.247	828	8.7%	-0.03 [-0.05, -0.01]	2015
Bhave 2016	-0.14	0.91202	145	-0.1	0.92039	107	1.1%	-0.04 [-0.27, 0.19]	2016
Bhave girl 2016	0.35	0.8088	159	0.15	0.7563	80	1.3%	0.20 [-0.01, 0.41]	2016
Xu 2017	-0.11	0.75	3733	0.03	0.66	3944	8.1%	-0.14 [-0.17, -0.11]	2017
Xu PA 2017	0.13	0.92	778	0.08	0.91	580	4.0%	0.05 [-0.05, 0.15]	2017
Xu NE 2017	0.05	0.88	792	0.08	0.91	580	4.1%	-0.03 [-0.13, 0.07]	2017
Rerksuppaphol 2017	0	0.918	111	0.45	0.8824	106	1.1%	-0.45 [-0.69, -0.21]	2017
Li 2019	-0.35	1.22	804	-0.23	1.34	777	2.9%	-0.12 [-0.25, 0.01]	2019
Liu 2019	0.06	0.7662	900	0.04	0.7779	939	5.5%	0.02 [-0.05, 0.09]	2019
Lin 2019	-0.01	0.8572	90	0.06	0.6752	107	1.2%	-0.07 [-0.29, 0.15]	2019
Habib-Mourad 2020	0.07	0.047	527	0.145	0.048	447	9.2%	-0.07 [-0.08, -0.07]	2020
Liang 2020	0	0.8591	29	0.1	0.7589	51	0.5%	-0.10 [-0.48, 0.28]	2020
**Subtotal (95% CI)**			**15943**			**15117**	**83.9%**	**-0.05 [-0.07, -0.02]**	
Heterogeneity: Tau² = 0.00; Chi² = 823.04, df = 15 (P < 0.00001); I² = 98%									
Test for overall effect: Z = 3.10 (P = 0.002)									
**Total (95% CI)**			**16730**			**15904**	**100.0%**	**-0.05 [-0.08, -0.03]**	
Heterogeneity: Tau² = 0.00; Chi² = 854.91, df = 19 (P < 0.00001); I² = 98%									
Test for overall effect: Z = 4.06 (P < 0.0001)									
Test for subgroup differences: Chi² = 2.45, df = 1 (P = 0.12), I² = 59.2%									

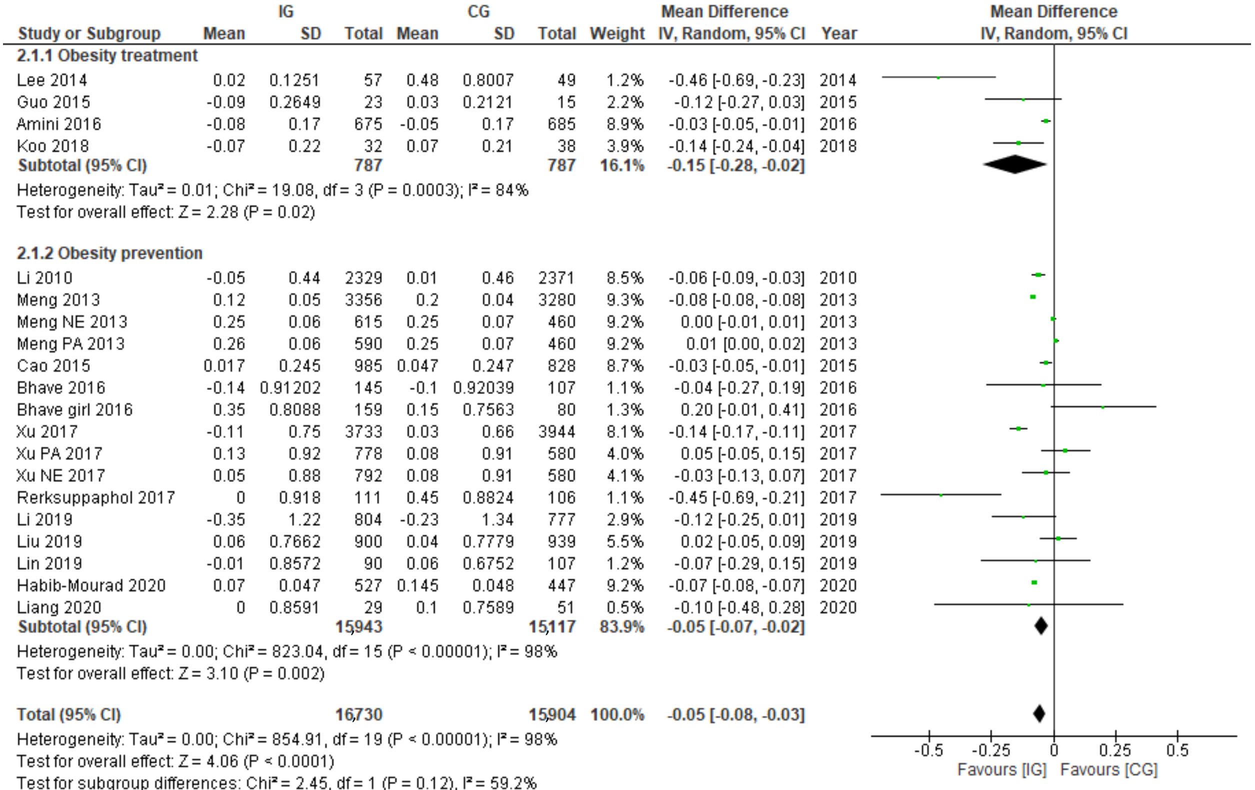

Figure S4. Pooled effects on BAZ, and the difference between overweight/obesity reduction and overweight/obesity prevention interventions

Study or Subgroup	IG Mean	IG SD	IG Total	CG Mean	CG SD	CG Total	Weight	Mean Difference IV, Random, 95% CI	Year
**1 Condcted by investigors**									
			0			0			
Heterogeneity: Not applicable									
Test for overall effect: Not applicable									
**2.1.2 Did not involve parents**									
Li 2010	-0.05	0.44	2329	0.01	0.46	2371	8.5%	-0.06 [-0.09, -0.03]	2010
Guo 2015	-0.09	0.2649	23	0.03	0.2121	15	2.2%	-0.12 [-0.27, 0.03]	2015
Bhave girl 2016	0.35	0.8088	159	0.15	0.7563	80	1.3%	0.20 [-0.01, 0.41]	2016
Bhave 2016	-0.14	0.91202	145	-0.1	0.92039	107	1.1%	-0.04 [-0.27, 0.19]	2016
Rerksuppaphol 2017	0	0.918	111	0.45	0.8824	106	1.1%	-0.45 [-0.69, -0.21]	2017
Liu 2019	0.06	0.7662	900	0.04	0.7779	939	5.5%	0.02 [-0.05, 0.09]	2019
Li 2019	-0.35	1.22	804	-0.23	1.34	777	2.9%	-0.12 [-0.25, 0.01]	2019
Habib-Mourad 2020	0.07	0.047	527	0.145	0.048	447	9.2%	-0.07 [-0.08, -0.07]	2020
Liang 2020	0	0.8591	29	0.1	0.7589	51	0.5%	-0.10 [-0.48, 0.28]	2020
**Subtotal (95% CI)**			**5027**			**4893**	**32.4%**	**-0.06 [-0.10, -0.02]**	
Heterogeneity: Tau² = 0.00; Chi² = 25.14, df = 8 (P = 0.001); I² = 68%									
Test for overall effect: Z = 3.02 (P = 0.002)									
**2.1.3 Involved parents**									
Meng 2013	0.12	0.05	3356	0.2	0.04	3280	9.3%	-0.08 [-0.08, -0.08]	2013
Meng NE 2013	0.25	0.06	615	0.25	0.07	460	9.2%	0.00 [-0.01, 0.01]	2013
Meng PA 2013	0.26	0.06	590	0.25	0.07	460	9.2%	0.01 [0.00, 0.02]	2013
Lee 2014	0.02	0.1251	57	0.48	0.8007	49	1.2%	-0.46 [-0.69, -0.23]	2014
Cao 2015	0.017	0.245	985	0.047	0.247	828	8.7%	-0.03 [-0.05, -0.01]	2015
Amini 2016	-0.08	0.17	675	-0.05	0.17	685	8.9%	-0.03 [-0.05, -0.01]	2016
Xu 2017	-0.11	0.75	3733	0.03	0.66	3944	8.1%	-0.14 [-0.17, -0.11]	2017
Xu PA 2017	0.13	0.92	778	0.08	0.91	580	4.0%	0.05 [-0.05, 0.15]	2017
Xu NE 2017	0.05	0.88	792	0.08	0.91	580	4.1%	-0.03 [-0.13, 0.07]	2017
Koo 2018	-0.07	0.22	32	0.07	0.21	38	3.9%	-0.14 [-0.24, -0.04]	2018
Lin 2019	-0.01	0.8572	90	0.06	0.6752	107	1.2%	-0.07 [-0.29, 0.15]	2019
**Subtotal (95% CI)**			**11703**			**11011**	**67.6%**	**-0.05 [-0.09, -0.01]**	
Heterogeneity: Tau² = 0.00; Chi² = 826.98, df = 10 (P < 0.00001); I² = 99%									
Test for overall effect: Z = 2.69 (P = 0.007)									
**Total (95% CI)**			**16730**			**15904**	**100.0%**	**-0.05 [-0.08, -0.03]**	
Heterogeneity: Tau² = 0.00; Chi² = 854.91, df = 19 (P < 0.00001); I² = 98%									
Test for overall effect: Z = 4.06 (P < 0.0001)									
Test for subgroup differences: Chi² = 0.07, df = 1 (P = 0.79), I² = 0%									

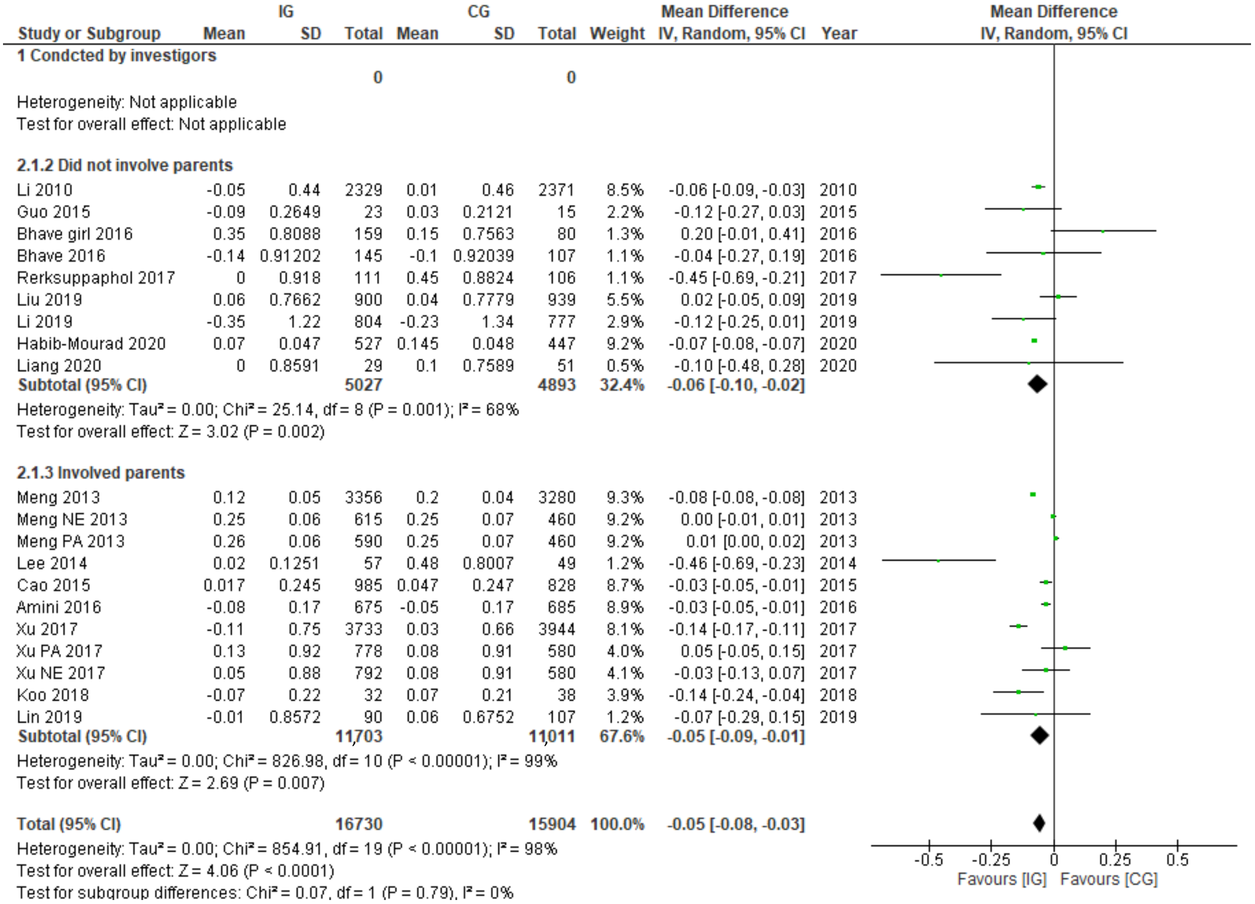

Figure S5. Pooled effects on BAZ, and the difference between interventions with parents' involvement and without parents' involvement

Study or Subgroup	IG Mean	IG SD	IG Total	CG Mean	CG SD	CG Total	Weight	Mean Difference IV, Random, 95% CI	Year
**2.1.2 With healthy food provision**									
Meng 2013	0.12	0.05	3356	0.2	0.04	3280	9.3%	-0.08 [-0.08, -0.08]	2013
Meng NE 2013	0.25	0.06	615	0.25	0.07	460	9.2%	0.00 [-0.01, 0.01]	2013
Cao 2015	0.017	0.245	985	0.047	0.247	828	8.7%	-0.03 [-0.05, -0.01]	2015
Bhave girl 2016	0.35	0.8088	159	0.15	0.7563	80	1.3%	0.20 [-0.01, 0.41]	2016
Bhave 2016	-0.14	0.91202	145	-0.1	0.92039	107	1.1%	-0.04 [-0.27, 0.19]	2016
Amini 2016	-0.08	0.17	675	-0.05	0.17	685	8.9%	-0.03 [-0.05, -0.01]	2016
Koo 2018	-0.07	0.22	32	0.07	0.21	38	3.9%	-0.14 [-0.24, -0.04]	2018
Liu 2019	0.06	0.7662	900	0.04	0.7779	939	5.5%	0.02 [-0.05, 0.09]	2019
Li 2019	-0.35	1.22	804	-0.23	1.34	777	2.9%	-0.12 [-0.25, 0.01]	2019
Habib-Mourad 2020	0.07	0.047	527	0.145	0.048	447	9.2%	-0.07 [-0.08, -0.07]	2020
**Subtotal (95% CI)**			**8198**			**7641**	**59.9%**	**-0.04 [-0.07, -0.01]**	
Heterogeneity: Tau² = 0.00; Chi² = 413.76, df = 9 (P < 0.00001); I² = 98%									
Test for overall effect: Z = 2.95 (P = 0.003)									
**2.1.3 Without**									
Li 2010	-0.05	0.44	2329	0.01	0.46	2371	8.5%	-0.06 [-0.09, -0.03]	2010
Meng PA 2013	0.26	0.06	590	0.25	0.07	460	9.2%	0.01 [0.00, 0.02]	2013
Lee 2014	0.02	0.1251	57	0.48	0.8007	49	1.2%	-0.46 [-0.69, -0.23]	2014
Guo 2015	-0.09	0.2649	23	0.03	0.2121	15	2.2%	-0.12 [-0.27, 0.03]	2015
Rerksuppaphol 2017	0	0.918	111	0.45	0.8824	106	1.1%	-0.45 [-0.69, -0.21]	2017
Xu 2017	-0.11	0.75	3733	0.03	0.66	3944	8.1%	-0.14 [-0.17, -0.11]	2017
Xu PA 2017	0.13	0.92	778	0.08	0.91	580	4.0%	0.05 [-0.05, 0.15]	2017
Xu NE 2017	0.05	0.88	792	0.08	0.91	580	4.1%	-0.03 [-0.13, 0.07]	2017
Lin 2019	-0.01	0.8572	90	0.06	0.6752	107	1.2%	-0.07 [-0.29, 0.15]	2019
Liang 2020	0	0.8591	29	0.1	0.7589	51	0.5%	-0.10 [-0.48, 0.28]	2020
**Subtotal (95% CI)**			**8532**			**8263**	**40.1%**	**-0.09 [-0.16, -0.03]**	
Heterogeneity: Tau² = 0.01; Chi² = 133.75, df = 9 (P < 0.00001); I² = 93%									
Test for overall effect: Z = 2.82 (P = 0.005)									
**Total (95% CI)**			**16730**			**15904**	**100.0%**	**-0.05 [-0.08, -0.03]**	
Heterogeneity: Tau² = 0.00; Chi² = 854.91, df = 19 (P < 0.00001); I² = 98%									
Test for overall effect: Z = 4.06 (P < 0.0001)									
Test for subgroup differences: Chi² = 1.96, df = 1 (P = 0.16), I² = 48.9%									

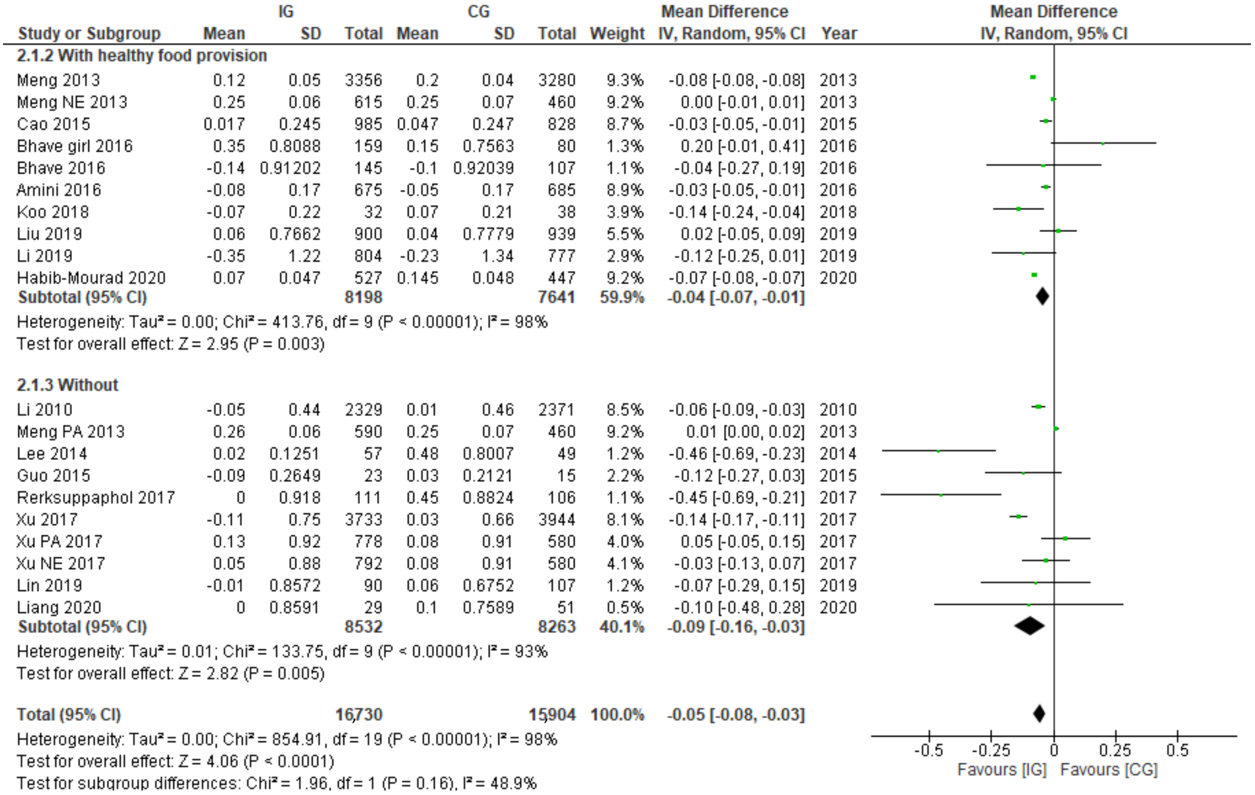

Figure S6. Pooled effects on BAZ, and the difference between interventions with healthy food provision and without

Study or Subgroup	IG Mean	IG SD	IG Total	CG Mean	CG SD	CG Total	Weight	Mean Difference IV, Random, 95% CI
**2.2.1 Multi-component**								
Guo 2015	0.77	1.5217	22	1.11	1.57778	13	0.0%	-0.34 [-1.41, 0.73]
hao NE PA boy 2019	-1.7	1.7418	31	0.1	1.5297	29	0.0%	-1.80 [-2.63, -0.97]
Hao NE PA girl 2019	-1.8	1.3416	25	0.2	1.2369	27	0.0%	-2.00 [-2.70, -1.30]
Jiang 2007	0.6	1.7624	1029	2.8	2.04	1396	13.4%	-2.20 [-2.35, -2.05]
Lee 2016	-0.6	2.0888	15	-0.1	1.6475	15	0.0%	-0.50 [-1.85, 0.85]
Liu 2019	0.73	2.2149	900	0.61	2.3202	939	12.0%	0.12 [-0.09, 0.33]
Meng 2013	0.65	0.09	3356	0.84	0.09	3280	15.6%	-0.19 [-0.19, -0.19]
Sevinc NE PA 2011	0.37	1.08	1932	0.51	0.98	2926	0.0%	-0.14 [-0.20, -0.08]
Shofan 2011	0.94	1.5	82	0.48	1.23	26	0.0%	0.46 [-0.11, 1.03]
Wang 2015	0.8	1.9228	90	1.04	1.8152	136	5.7%	-0.24 [-0.74, 0.26]
Xu 2015	-0.32	1.36	605	-0.29	1.4	503	0.0%	-0.03 [-0.19, 0.13]
Xu 2017	0.6	4.8109	3476	0.8	4.8266	3398	0.0%	-0.20 [-0.43, 0.03]
**Subtotal (95% CI)**			**5375**			**5751**	**46.7%**	**-0.63 [-1.74, 0.47]**
Heterogeneity: Tau² = 1.25; Chi² = 681.56, df = 3 (P < 0.00001); I² = 100%								
Test for overall effect: Z = 1.12 (P = 0.26)								
**2.2.2 Single component (NE or Exercise)**								
Hao NE boy 2019	-1.3	1.5601	33	0.1	1.5297	29	0.0%	-1.40 [-2.17, -0.63]
Hao NE girl 2019	-0.7	1.4401	27	0.2	1.2369	27	0.0%	-0.90 [-1.62, -0.18]
Hao PA boy 2019	-1.7	1.6787	32	0.1	1.5297	29	0.0%	-1.80 [-2.61, -0.99]
Hao PA girl 2019	-1.7	1.5601	25	0.2	1.2369	27	0.0%	-1.90 [-2.67, -1.13]
Li 2010	0.56	1.15	2329	0.72	1.2	2371	15.1%	-0.16 [-0.23, -0.09]
Lin 2019	0.03	2.7484	90	0.21	2.0885	107	3.6%	-0.18 [-0.87, 0.51]
Meng NE 2013	0.74	0.15	615	0.72	0.15	460	15.5%	0.02 [0.00, 0.04]
Meng PA 2013	0.76	0.15	590	0.72	0.15	460	15.5%	0.04 [0.02, 0.06]
Rerksuppaphol 2017	0.14	11.6556	111	1.04	10.8278	106	0.0%	-0.90 [-3.89, 2.09]
Sevinc NE 2011	0.35	1.13	1989	0.51	0.98	2926	0.0%	-0.16 [-0.22, -0.10]
Toruner 2010	-0.3	1.2649	41	0	1.2649	40	0.0%	-0.30 [-0.85, 0.25]
wang NE 2015	1.4	4.2298	96	1.04	4.0467	136	1.7%	0.36 [-0.73, 1.45]
wang PA 2015	1.03	4.279	116	1.04	4.0467	136	1.9%	-0.01 [-1.04, 1.02]
Xu NE 2017	0.7	4.1023	628	0.7	3.7475	466	0.0%	0.00 [-0.47, 0.47]
Xu PA 2017	0.8	4.3192	605	0.7	3.7475	466	0.0%	0.10 [-0.38, 0.58]
**Subtotal (95% CI)**			**3836**			**3670**	**53.3%**	**-0.02 [-0.07, 0.04]**
Heterogeneity: Tau² = 0.00; Chi² = 32.63, df = 5 (P < 0.00001); I² = 85%								
Test for overall effect: Z = 0.62 (P = 0.53)								
**Total (95% CI)**			**9211**			**9421**	**100.0%**	**-0.34 [-0.49, -0.19]**
Heterogeneity: Tau² = 0.04; Chi² = 1706.07, df = 9 (P < 0.00001); I² = 99%								
Test for overall effect: Z = 4.43 (P < 0.00001)								
Test for subgroup differences: Chi² = 1.18, df = 1 (P = 0.28), I² = 15.6%								

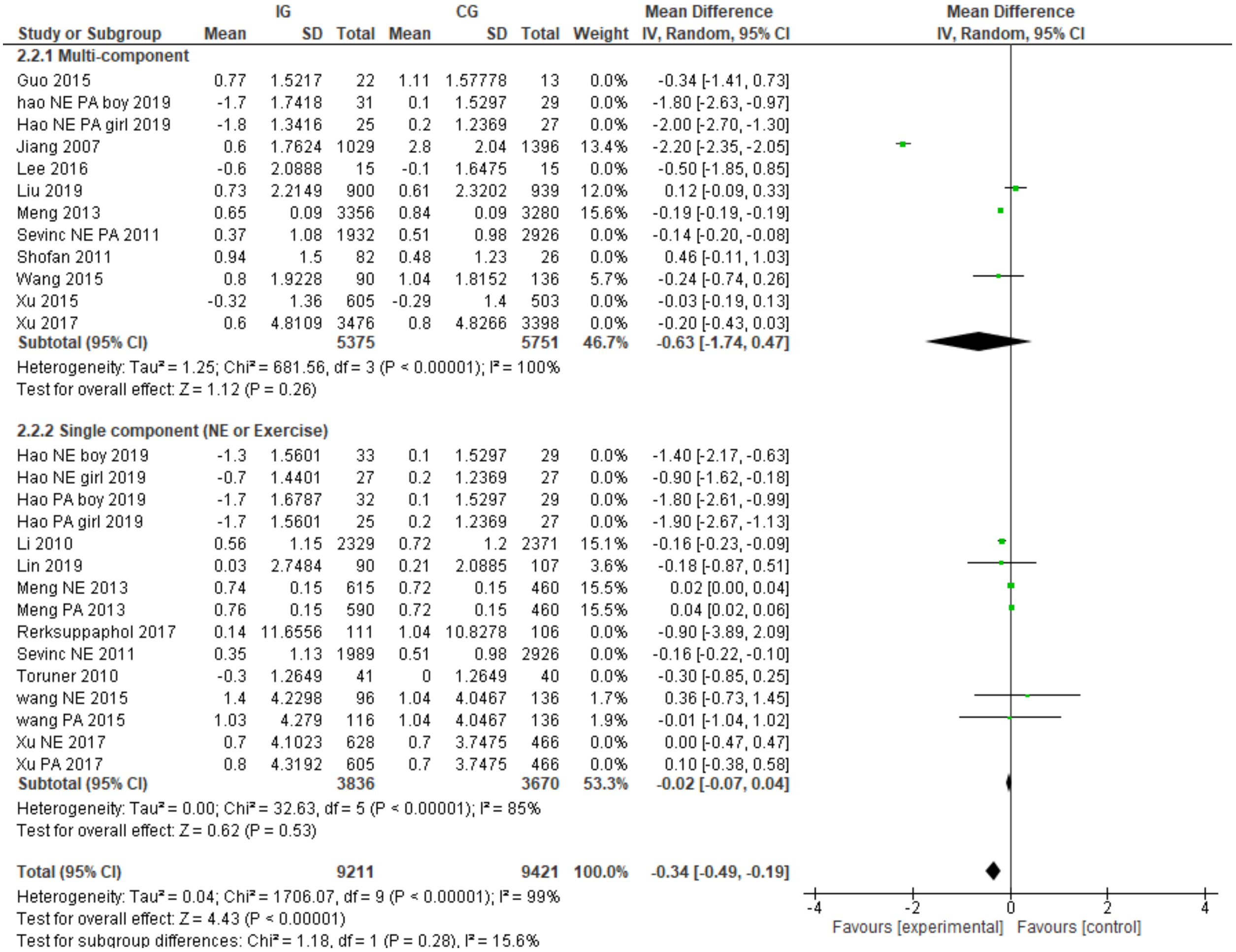

Figure S7. Sensitivity analysis: removing high risk of bias studies

Study or Subgroup	IG Mean	IG SD	IG Total	CG Mean	CG SD	CG Total	Weight	Mean Difference IV, Random, 95% CI
**2.2.1 Multi-component**								
Guo 2015	0.77	1.5217	22	1.11	1.57778	13	0.9%	-0.34 [-1.41, 0.73]
hao NE PA boy 2019	-1.7	1.7418	31	0.1	1.5297	29	1.4%	-1.80 [-2.63, -0.97]
Hao NE PA girl 2019	-1.8	1.3416	25	0.2	1.2369	27	1.9%	-2.00 [-2.70, -1.30]
Jiang 2007	0.6	1.7624	1029	2.8	2.04	1396	7.3%	-2.20 [-2.35, -2.05]
Lee 2016	-0.6	2.0888	15	-0.1	1.6475	15	0.0%	-0.50 [-1.85, 0.85]
Liu 2019	0.73	2.2149	900	0.61	2.3202	939	6.5%	0.12 [-0.09, 0.33]
Meng 2013	0.65	0.09	3356	0.84	0.09	3280	8.6%	-0.19 [-0.19, -0.19]
Sevinc NE PA 2011	0.37	1.08	1932	0.51	0.98	2926	8.3%	-0.14 [-0.20, -0.08]
Shofan 2011	0.94	1.5	82	0.48	1.23	26	0.0%	0.46 [-0.11, 1.03]
Wang 2015	0.8	1.9228	90	1.04	1.8152	136	0.0%	-0.24 [-0.74, 0.26]
Xu 2015	-0.32	1.36	605	-0.29	1.4	503	7.2%	-0.03 [-0.19, 0.13]
Xu 2017	0.6	4.8109	3476	0.8	4.8266	3398	6.2%	-0.20 [-0.43, 0.03]
**Subtotal (95% CI)**			**11376**			**12511**	**48.4%**	**-0.67 [-1.02, -0.33]**
Heterogeneity: Tau² = 0.23; Chi² = 728.10, df = 8 (P < 0.00001); I² = 99%								
Test for overall effect: Z = 3.82 (P = 0.0001)								
**2.2.2 Single component (NE or Exercise)**								
Hao NE boy 2019	-1.3	1.5601	33	0.1	1.5297	29	1.6%	-1.40 [-2.17, -0.63]
Hao NE girl 2019	-0.7	1.4401	27	0.2	1.2369	27	1.8%	-0.90 [-1.62, -0.18]
Hao PA boy 2019	-1.7	1.6787	32	0.1	1.5297	29	1.5%	-1.80 [-2.61, -0.99]
Hao PA girl 2019	-1.7	1.5601	25	0.2	1.2369	27	1.6%	-1.90 [-2.67, -1.13]
Li 2010	0.56	1.15	2329	0.72	1.2	2371	8.3%	-0.16 [-0.23, -0.09]
Lin 2019	0.03	2.7484	90	0.21	2.0885	107	1.9%	-0.18 [-0.87, 0.51]
Meng NE 2013	0.74	0.15	615	0.72	0.15	460	8.5%	0.02 [0.00, 0.04]
Meng PA 2013	0.76	0.15	590	0.72	0.15	460	8.5%	0.04 [0.02, 0.06]
Rerksuppaphol 2017	0.14	11.6556	111	1.04	10.8278	106	0.1%	-0.90 [-3.89, 2.09]
Sevinc NE 2011	0.35	1.13	1989	0.51	0.98	2926	8.3%	-0.16 [-0.22, -0.10]
Toruner 2010	-0.3	1.2649	41	0	1.2649	40	2.7%	-0.30 [-0.85, 0.25]
wang NE 2015	1.4	4.2298	96	1.04	4.0467	136	0.0%	0.36 [-0.73, 1.45]
wang PA 2015	1.03	4.279	116	1.04	4.0467	136	0.0%	-0.01 [-1.04, 1.02]
Xu NE 2017	0.7	4.1023	628	0.7	3.7475	466	3.3%	0.00 [-0.47, 0.47]
Xu PA 2017	0.8	4.3192	605	0.7	3.7475	466	3.2%	0.10 [-0.38, 0.58]
**Subtotal (95% CI)**			**7115**			**7514**	**51.6%**	**-0.13 [-0.21, -0.04]**
Heterogeneity: Tau² = 0.01; Chi² = 129.68, df = 12 (P < 0.00001); I² = 91%								
Test for overall effect: Z = 2.94 (P = 0.003)								
**Total (95% CI)**			**18491**			**20025**	**100.0%**	**-0.39 [-0.50, -0.28]**
Heterogeneity: Tau² = 0.04; Chi² = 1801.04, df = 21 (P < 0.00001); I² = 99%								
Test for overall effect: Z = 7.03 (P < 0.00001)								
Test for subgroup differences: Chi² = 9.02, df = 1 (P = 0.003), I² = 88.9%								

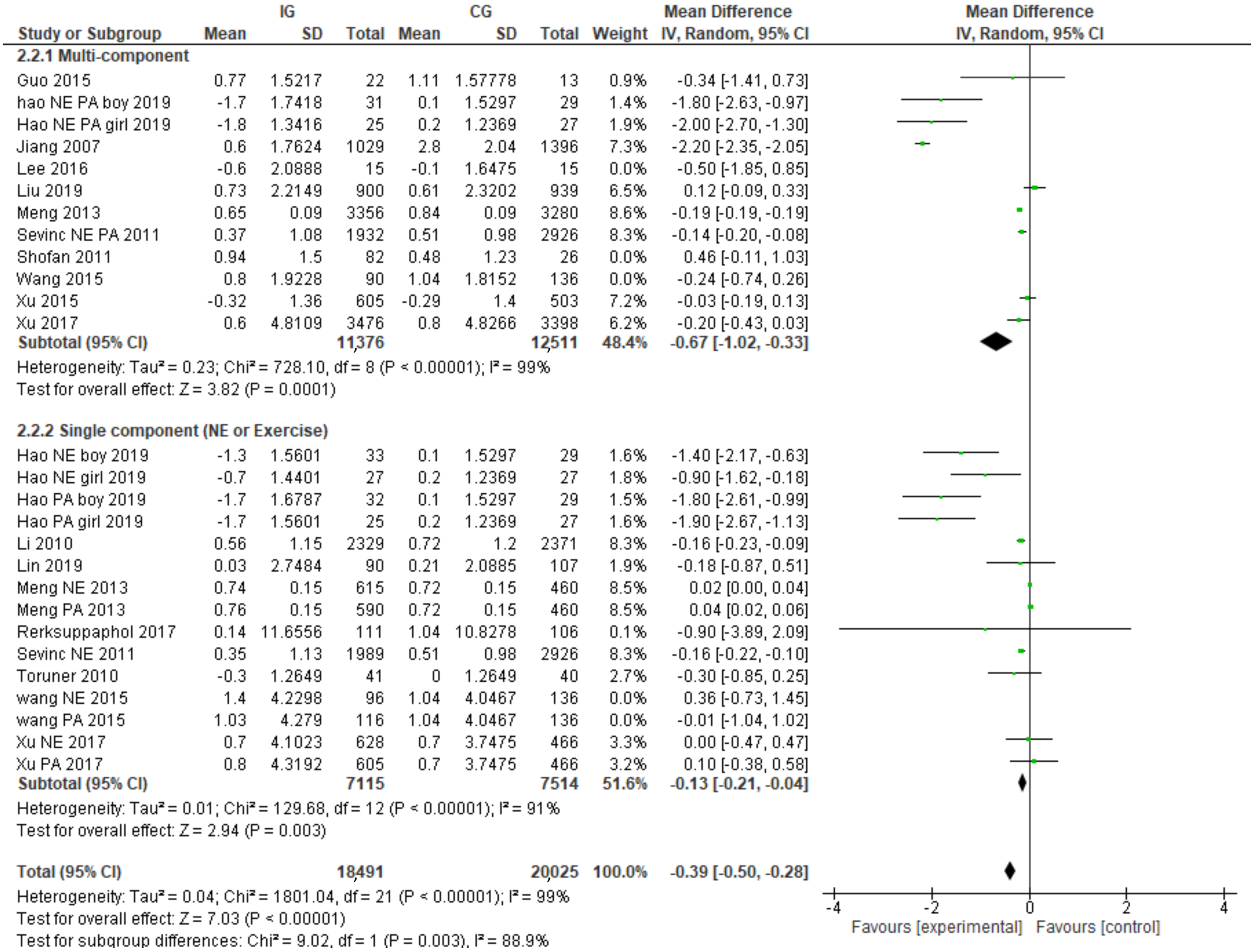

Figure S8. Sensitivity analysis: removing quasi-experimental studies

Table S1. Characteristics of studies included in this review

Reference	Title	Objective	Study design	Sample
Akdemir M, et al, 2017	The Effect of Nutritional and Physical Activity Interventions on Nutritional Status and Obesity in Primary School Children: A Cluster Randomized Controlled Study	To measure the effect of intervention on preventing and reducing obesity by teaching healthy nutritional behavior and physical activity among primary school children	Cluster RCT	**Intervention:** 1 primary school, grade 1-8, 675 students **Control:** 1 school, grade 1-8, 685 students
Amini M, et al, 2016	A School-Based Intervention to Reduce Excess Weight in Overweight and Obese Primary School Students	Evaluated the effect of an intervention for reducing excess weight gain in primary school-age children in Tehran	Cluster RCT	**Intervention:** 6 schools, grade 4-6, 167 overweight students **Control:** 6 schools, grade 4-6, 167 overweight students
Aperman-Itzhak, T, et al, 2004	School-Based Intervention to Promote a Healthy Lifestyle and Obesity Prevention Among Fifth- and Sixth-Grade Children	To evaluate the effectiveness of a healthy lifestyle intervention on health knowledge, behavior, and anthropometric measurements	Quasi experiments	**Intervention:** 2 religious and 2 secular schools (not BW selective), grade 5-6, 200 students **Control:** 2 religious and 2 secular schools (no weight selective), grade 5-6, 196 students (matched by sociodemographic characteristics and religious status)
Bhave S, et al, 2016	Effectiveness of a 5-year school-based intervention programme to reduce adiposity and improve fitness and lifestyle in	To report the effectiveness of a non-randomised intervention carried out in one school in	Quasi experiments	**Intervention:** Symbiosis Pune school, grade 3-4 students, 375 students

Reference	Title	Objective	Study design	Sample
	Indian children; the SYM-KEM study	the city of Pune in western India.		**Control:** Symbiosis Nasik school (200 km away), 1. grade 3-4 students from: 209 students, 2. grade 8-9 students from the same school as IG group (compare their 2005-2006 data to the IG's 2010 -2011 data): 374 students
El Harake MD, et al, 2018	Impact of a pilot school-based nutrition intervention on dietary knowledge, attitudes, behavior and nutritional status of Syrian refugee children in the Bekaa, Lebanon.	To evaluate the impact of a 6-month school nutrition intervention on changes in dietary knowledge, attitude, behavior (KAB) and nutritional status of Syrian refugee children	Quasi experiments	**Intervention:** 2 informal schools, Syrian refugee children grade 4 - 6, 195 students **Control:** 1 informal school, Syrian refugee grade 4-6, 101 students
Habib-Mourad C, et al, 2020	Impact of a three-year obesity prevention study on healthy behaviors and BMI among Lebanese schoolchildren: Findings from Ajyal Salima Program	To investigate (1) the long-term effects of a school-based intervention program when implemented over two years on body mass index (BMI), healthy dietary behaviors, and physical activity (PA); and (2) whether the effects are sustained after one-year washout.	Cluster RCT	**Intervention:** public & private schools (different SES), grade 4-5, 698 students **Control:** public & private schools (different SES), grade 4-5, 541 students
Hao M, et al, 2019	Short-Term and Long-Term Effects of a Combined Intervention of Rope Skipping and Nutrition Education for Overweight Children in Northeast China	To evaluate whether an exercise intervention, nutrition education, or the combination of both were effective in weight reduction and	Quasi experiments	**Intervention:** grade 4-6 overweight students, PA group 57 students, nutrition

Reference	Title	Objective	Study design	Sample
		maintenance for rural school children		education 60, PA & Nutrition education 56 students **Control:** grade 4-6 overweight students, 56 students
Jiang J, et al, 2007	The effects of a 3-year obesity intervention in schoolchildren in Beijing	To measure the effects of a primary school-based intervention programme on the prevalence of obesity in Beijing	Clustered RCT	**Intervention:** 2 primary schools, 1029 students **Control:** 3 primary schools, (matched by school size, physical education and socio-economic status), 1396 students
Koo HC, et al 2018	The GReat-Child Trial: A Quasi-Experimental Intervention on Whole Grains with Healthy Balanced Diet to Manage Childhood Obesity in Kuala Lumpur, Malaysia	To test the hypothesis that a whole grains intervention for the treatment of childhood obesity would have a greater effect on anthropometric measurements	Quasi experiments	**Intervention:** 40 overweight students **Control:** 43 overweight students
Lee A, et al, 2014	Childhood obesity management shifting from health care system to school system: intervention study of school-based weight management programme	Evaluation of the effectiveness of a multi-component school-based weight management programme for overweight and obese primary school children via a home-school joint venture	RCT	**Intervention:** 57 overweight students **Control:** 49 overweight students
Lin YC, et al, 2019	NASA Mission X Program for Healthy Eating and Active Living among Taiwanese Elementary School Students	Assessed the effects of an intervention program adapted from the NASA Mission X (MX) program	Cluster RCT	**Intervention:** 4 schools, grade 3-4,92 students **Control:** 4 schools, grade 3-4, 109 students

Reference	Title	Objective	Study design	Sample
		on children's Healthy Eating Active Living (HEAL) knowledge and behaviors and anthropometry		
Meng LP, et al, 2013	The Costs and Cost-Effectiveness of a School-Based Comprehensive Intervention Study on Childhood Obesity in China	To evaluate the cost and the cost-effectiveness of a comprehensive intervention program for childhood obesity.	Multi-center RCT	**Intervention:** non-boarding school with >=10% overweight & >50% have lunch at school, grade 1-5, Beijing: Nutrition education 615, PA 590, Other 5 cities: Nutrition education & PA 3356 **Control:** Beijing: 460. Other 5 cities: 3280
Rerksuppaphol L & Rerksuppaphol S, 2017	Internet Based Obesity Prevention Program for Thai School Children- A Randomized Control Trial	To assess the efficacy of internet-based obesity prevention program in Thai school children	RCT	**Intervention:** 1 school, grade 1-6, 111 students with BMI >=-1 SD, no NCD, can stand straight **Control:** 1 school, grade 1-6, 107 students
Sevinc O, et al, 2011	Evaluation of the effectiveness of an intervention program on preventing childhood obesity in Denizli, Turkey	To determine the effi ciency of 2 different intervention programs (healthy nutrition education and/or physical activity programs) for preventing the obesity of primary school students	Cluster RCT	**Intervention:** Grade 1-7, PA & Nutrition education: 1932 students, Nutrition education: 1989 students **Control:** Grade 1-7, 2926 students
Toruner EK, et al, 2015	Efficacy of a School-Based Healthy Life Program in Turkey	To evaluate the effectiveness of a	Cluster RCT	**Intervention:** from 2 schools, grade 3-7,497 students

Reference	Title	Objective	Study design	Sample
		school-based healthy life program		**School:** 2 schools, grade 3-7, 470 students
Wang JJ, et al, 2015	Evaluation of a comprehensive intervention with a behavioural modification strategy for childhood obesity prevention: a nonrandomized cluster-controlled trial	Describes the development and implementation of a comprehensive, social cognitive behaviour modification intervention using accelerometry and a dietary diary to tackle child overweight and obesity	Quasi experiments	**Intervention:** overweight children aged 7-12 yr old: G1 .PA+diet (school a) 90 students, G2.PA (school B C D) 116 students, G3 diet (school E F G) 96 students **Control:** School (H I J) 136 students
Xu HQ, 2017	Comprehensive school-based intervention to control overweight and obesity in China: a cluster randomized controlled trial	To evaluate the effect of comprehensive school-based intervention on childhood obesity	Multi-center cluster RCT	**Intervention:** 6-13 years, 15 schools, 4827 students **Control:** 6-13 years, 17 schools, 4026 students
Cao ZJ, et al, 2015	A Randomized Trial of Multiple Interventions for Childhood Obesity in China	To evaluate the effectiveness of a family-individual-school–based comprehensive intervention model.	cluster RCT	**Intervention:** 8 schools, 1287 first graders **Control:** 9 schools, 1159 first graders
Guo T, et al, 2015	Intervention of childhood and adolescents obesity in Shantou city	To evaluate the effects of multicomponent school-based intervention constituted of diet modification, regular exercise and psychosocial consultation on body status in overweight and obese children and adolescents. And to come up with an appropriate intervention protocol	cluster RCT	**Intervention:** 3-5 graders, 26 overweight/obese **Control:** 3-5 graders,15 overweight/obese

Reference	Title	Objective	Study design	Sample
		for controlling children and adolescents' obesity in Shantou city		
Shofan Y, et al, 2011	A school-based program of physical activity may prevent obesity	To evaluate the effects of a 2-year intervention program in elementary school on the prevention of obesity	quasi-experiment	**Intervention:** 82 children aged 9-11 yrs **Control:** 27 children aged 9-11 yrs
Lee GY, et al, 2016	Effects of an obesity management mentoring program for Korean children	To develop and test a mentored obesity management program guiding physical exercise, improving eating habits, and promoting self-esteem among elementary school learners.	quasi-experiment	**Intervention:** Overweight/obese 9-12 yrs old, 17 students **Control:** Overweight/obese 9-12 yrs old, 15 students
Li B, et al,	The CHIRPY DRAGON intervention in preventing obesity in Chinese primary-school--aged children: A cluster-randomised controlled trial	To evaluate clinical- and cost- effectiveness of the Chinese Primary School Children Physical Activity and Dietary Behaviour Changes Intervention (CHIRPY DRAGON) developed using the UK MRC complex intervention framework to prevent obesity in Chinese primary-school–aged children	Cluster RCT	**Intervention:** 20 schools, 832 students aged 6 years **Control:** 20 schools, 809 students aged 6 years
Li YP, et al, 2010	Report on childhood obesity in China (8): Effects and sustainability of physical activity intervention on body composition of Chinese youth	To determine whether a large-scale physical activity intervention could affect body composition in primary school students in Beijing, China	Cluster RCT	**Intervention:** 10 schools, 2371 students (grades 3-4) **Control:** 10 schools, 2329 students (grades 3-4)
Liu A, et al, 2008	Evaluation of a classroom-based	To evaluate the effect of the Happy 10 programme on the	quasi-experiment	**Intervention:** 1 school, grades 1-5, 328 students

Reference	Title	Objective	Study design	Sample
	physical activity promoting programme	promotion of physical activity, physical growth and development of primary school students, and on obesity control and prevention		**Control:** 2 school, grades 1-5, 425 students
Liu Z, et al, 2019	A School-Based Comprehensive Intervention for Childhood Obesity in China: A Cluster Randomized Controlled Trial	To evaluate a theory-based comprehensive intervention implemented within primary schools for childhood obesity in China	cluster RCT	**Intervention:** 6 schools, 930 students aged 7-11 yrs old **Control:** 7 schools, 959 students aged 7-11 yrs old
Toruner EK & Savaser S, 2010	A Controlled Evaluation of a School-Based Obesity Prevention in Turkish School Children	To assess the effect of a weight management program in Turkish school children with overweight and obesity	RCT	**Intervention:** 1 school, grade 4, 41 overweight/obese students **Control:** 1 school, grade 4, 40 overweight/obese students
Xu F, et al, 2015	Effectiveness of a Randomized Controlled Lifestyle Intervention to Prevent Obesity among Chinese Primary School Students: CLICK-Obesity Study	To assess the effectiveness of a school-based multi-component lifestyle childhood obesity prevention program (the CLICK Obesity study) in Mainland China.	Cluster RCT	**Intervention:** 4 schools, grade 4, 638 students **Control:** 4 schools, grade 4, 544 students

Table S2. Intervention characteristics

Reference	Adoption	Component & delivery method	Duration	Context	Outcome	Maintenance
Akdemir M et al, 2017.	• Introduction of the intervention<br>Investigators provided nutrition education to parents to encourage a healthy diet and active lifestyle in children.<br><br>• Adoption of stakeholders<br>No information on the extent of adoption from the schools or parents.	1. Extra nutrition education (ENE) for students by an investigator<br>5 sessions of a 40-minute class about "healthy diet & active lifestyle" and "obesity prevention" (3 times before and 2 after school breaks).<br>2. Nutrition education for parents by an investigator.<br>2 sessions of a 1-hour workshop and 1 brochure.	1 school year	A city with higher SES and better health outcomes than the average of Turkey (an upper-middle income & central Asia country)	1.04-fold increase of the prevalence of normal weight students in the intervention group compared to the control group n (RR = 1.04; 95% CI = 1.01 - 1.06; p = 0.0025)	NA
Amini M, et al, 2016.	• Introduction of the intervention<br>1. Face-to-face training and a guidebook for school health instructors to provide nutrition education to students<br>2. Investigators provided lifestyle education for parents<br>• Adoption of stakeholders<br>Teachers' collaborations varied across schools. Parents attended the meetings for 40%.	1. ENE for students<br>12 weekly sessions of 15-45 minutes classes (food groups & body weight management) by school health instructors.<br>2. Lifestyle education for parents<br>4 monthly sessions of 20-minute healthy lifestyle sessions by investigators.<br>3. Extra PA education for students (2 hrs/week).<br>4. Schools' canteens improvement asked staff to stop selling high-calorie food, reduce oil use, and increase whole grains and vegetables (monitored the change).	18 weeks	Low- and middle-class districts of urban capital city of Iran (an upper middle-income, west Asia country)	• Mean differences of BMI z score in the intervention group vs the control group were (-0.08) $\pm$ 0.17 vs (-0.05) $\pm$ 0.17, $p<0.05$.	NA
Aperman-Itzhak T, et al, 2004.	• Introduction of the intervention	1. Nutrition education for students	1 school year	A city with different religions and mixed	In the intervention group, overweight and obesity decreased	NA

Reference	Adoption	Component & delivery method	Duration	Context	Outcome	Maintenance
	1. Collaborated with the local council to gain full support from the local stakeholders.<br>2. Launched opening ceremony-"healthy year" with a city march.<br>3. The intervention was delivered by teachers.<br>• Adoption of stakeholders<br>No information on the extent of adoption from the stakeholders.	Teachers integrated healthy eating and physical activity contents in different subjects according to the MOE guidance.<br>2. PA promotion in schools<br>Teacher led active breaks & schools provided attractive playgrounds.<br>3. teacher encouraged healthy snacks eating.<br>4. City campaign "A happy year"<br>5. Nutrition education for parents Label reading & healthy eating plan<br>6. PA promotion in community<br>Leisure activity places discounted fares		cultures in Israel (a high-income and central Asia country).	from 25% to 17.9%, $p<0.05$). In the control group, no significant change was found (from 20.5% to 17.6%, $p = 0.12$).	
El Harake MD, et al, 2018.	• Introduction of the intervention<br>Two 2-day workshops (prior and refreshing) for teachers. One workshop for kitchen staff.<br>• Adoption of stakeholders<br>Good adherence to the intervention components.	1. ENE for students<br>Teachers provided interactive classes for 45 mins bi-weekly basis in 6 months (hygienic practices, importance of consuming breakfast daily, role of fruits and vegetables in a healthy diet, benefits of consuming water versus sugar-sweetened beverages, healthy snacking behaviors, and importance of physical activity) using hands-on activities and games, visually appealing and culturally sensitive posters and printed material technique.<br>2. Provision of locally prepared healthy snacks (357 kcal per	2 school years	Informal schools for refugees in underserved rural Bekaa region of Lebanon (an upper middle-income and central Asia country)	Improved undernutrition<br>• Mean difference of BMI for-age-z score in the intervention group = 0.10 $\pm$ 0.06, while in the control group = (-0.10 $\pm$ 0.08, $p=0.039$)<br>• Increased BMI for-age-z score in the intervention group ($\beta = 0.25$, 95% CI = 0.10, 0.41).	NA

Reference	Adoption	Component & delivery method	Duration	Context	Outcome	Maintenance
		day, 11 g protein, 58 g carbohydrates, and 9 g of fat).				
Hao M, et al, 2019.	• Introduction of the intervention<br>A local nutritionist provided nutrition education. Teachers provided exercise intervention (skipping rope).<br>• Adoption of stakeholders<br>No information on the extent of adoption from the teachers.	Group1. PA<br>30 minutes of rope skipping/day & two 45-minute physical education classes/week by teachers.<br>Group2. ENE<br>45 min class 2 times/week for 2 months (growth of children, nutritional requirement for children, daily diet plan, healthy eating habits, PA, health) by a local nutritionist.<br>Group3. PA & ENE	2 months	Rural elementary schools with the largest number of students in Benxi City, Liaoning Province, in Northeast China (an upper middle-income and east Asia country)	Mean BMI in all the interventions (PA, ENE, PA&ENE), significantly decreased at the end of intervention compared to baseline, while mean BMI of the control group did not decreased. Among the interventions, PA&ENE provided the strongest effect, followed by PA.	Mean BMI in all the intervention groups (PA, ENE, PA&ENE), significantly decreased at 1-year follow-up compared to baseline, while mean BMI of the control group did not decreased. Among the interventions, PA&ENE provided the strongest effect, followed by ENE.
Jiang J, et al, 2007.	• Introduction of the intervention<br>Investigators trained teachers to teach nutrition class and a textbook was provided.<br>• Adoption of stakeholders<br>No information on the extent of adoption from the teachers.	1. ENE for students<br>by investigators<br>2. Nutrition education for parents<br>1 session/term by investigators about obesity, food pyramid, Chinese food composition tables, and a healthy lifestyle (vegetables and fruit consumption, 'traffic light' food item system, overeating, eating out in restaurants, fast food consumption, television viewing, computer games, and PA).	3 years	Beijing urban area in China	The overweight & prevalence in the intervention schools vs control schools: 9.8% vs. 14.4%, P < 0.01.<br>The obesity prevalence in the intervention schools vs control schools: 7.9% vs. 13.3%, P < 0.01. The prevalence of overweight and obesity decreased by 26.3%<br>and 32.5% in intervention schools,	NA

Reference	Adoption	Component & delivery method	Duration	Context	Outcome	Maintenance
		3. An extra meeting for parents with overweight and obese children<br>4. PA for overweight and obese children and students who failed PE tests<br>20-min run X 4 days/week,			respectively. The prevalence of overweight and obesity increased in control schools.	
Koo HC, et al, 2018.	• Introduction of the intervention<br>Investigators provided nutrition class to students and an individual consultation to parents.<br>• Adoption of stakeholders<br>No information on the extent of adoption from the parents.	1. ENE for students<br>30-min nutrition education classes X 6 times, which employed Food Guide Pyramid, visual plate model, whole grain food recommendation, and balanced diet<br>2. school delivery of whole-grain foods<br>food delivery to schools consisting of whole-grain bread, whole-grain biscuits, and whole-grain ready-to-eat cereal, on a daily basis of for 12 weeks.<br>3. Nutrition education for parents<br>1-hour individual meeting with parents to encourage the students to consume wholegrain food and to practice a balanced diet at home.	3 months	Keramat zone in Kuala Lumpur, Malaysia (an upper middle-income and southeast Asia country)	The intervention group had lower BMI for age z score compared to the control group at 9-month follow-up: −0.12; 95% CI: −0.21, −0.03; p = 0.009. There was no significant change of BMI for age z score among the intervention group (mean difference: −0.07 (−0.15, 0.01) p=0.092), while there was a significant increase among the control group (mean difference: 0.07 (0.01, 0.14), p=0.032) at the end of the intervention.<br>Waist circumference of the intervention group decreased (mean difference: −2.1 (−3.7, −0.5), p=0.014, while there was no change among the control group (mean	There was no significant change of BMI for age z score among the intervention group (mean difference: −0.06 (−0.25, 0.13), p=0.544), while there was a significant increase among the control group (mean difference: 0.18 (0.10, 0.26), p<0.001) at nine month later.<br>There was no significant change of waist circumference of the intervention group (mean difference: −1.9 (−4.1, 0.3), p=0.091), while there was an increase among the control group (mean difference:

Reference	Adoption	Component & delivery method	Duration	Context	Outcome	Maintenance
					difference: 0.7 (−0.3, 1.7), p=0.165)	2.5 (0.9, 4.1), p=0.002)
Lee A, et al, 2014.	• Introduction of the intervention<br>1. The project team (a dietician, a nutritionist and physiotherapist) guided the schools' teachers to implement the intervention in the schools<br>2. The project team worked with parents to encourage a healthy lifestyle in students<br>• Adoption of stakeholders<br>No information on the extent of adoption from the stakeholders.	Health Promoting School (HPS) approach<br>1. ENE, PA and self-image sessions for students<br>75-minute afterschool sessions and 3-hour weekend sessions<br>2. Nutrition education for parents<br>- An introductory seminar on the basic principles on weight management<br>-A 1-hour follow-up training on healthy eating and exercise strategies to assist weight control in children.<br>3. Label education training for parents and children.<br>4. Progress monitoring and solutions finding with parents<br>Discussed with parents about the progress, obstacles, and solutions.	4 months	Urban areas in Hong Kong-China (a high-income special administrative region of China)	The intervention group had lower BMI for age z score and body fat compared to the control group at the end of the intervention (BMI for age z-score: −0.21, 95% CI −0.34 to −0.07, P = 0.003), body fat: −2.67%, 95% CI −5.12 to −0.22, P = 0.033).	The intervention group had lower BMI for age z score compared to the control group at 4-month follow-up (− −0.06, 95% CI −0.11, −0.007, P = 0.028)
Rerksuppaphol L & Rerksuppaphol S, 2017.	• Introduction of the intervention<br>Investigators introduce a computer programme to students for self-learning. Teachers were asked to measure students' weights and heights and enter the data into the computer programme monthly.	Provision of a self-learning and self-monitoring computer programme for students<br>- Nutrition knowledge (nutritional status, recommendation of daily amounts of portion and serving sizes of each food groups, nutritional status)<br>- Physical activity recommendations based on students' nutritional status	4 months	A rural town in Thailand (an upper middle-income and southeast Asia country)	Prevalence of overweight and obesity in the intervention group vs the control group: 39.6% vs 56.6%, p =0.009. Net BMI gains in the intervention group vs the control group: 0.40kg/m2 vs 1.24kg/m2, p =0.027). The intervention	NA

Reference	Adoption	Component & delivery method	Duration	Context	Outcome	Maintenance
	• Adoption of stakeholders<br>No information on the extent of adoption from the stakeholders.				group had no changes in BMI for age z-score (-0.001, 95%CI -0.19 to 0.18, p =0.988), while the control group had increased BMI for age z-score at the end of study (0.45, 95%CI 0.27 to 0.63, p <0.001)	
Sevinc O, et al, 2011.	• Introduction of the intervention<br>Teachers were asked to add extra physical education classes. Investigators provided workshops for teachers and parents to encourage a healthy diet and body weight management in students.<br>• Adoption of stakeholders<br>No information on the extent of adoption from the stakeholders.	1. Extra physical education (PE) classes<br>Increased PE sessions from 2 h/wk to 3 h/wk.<br>2. Nutrition education for students<br>Workshops about healthy diet and obesity prevention by staff of the Health Training Division of the City Health Administration.<br>3. Nutrition education for parents and teachers<br>Workshops about healthy diet and childhood obesity prevention by staff of the Health Training Division of the City Health Administration.<br>4. Provision of boxed milks<br>5. Provision of healthier food in school canteens<br>e.g., water, freshly squeezed fruit juice, buttermilk, milk,	8 months	School having a half-day education system, located in low- and high SES regions in an industrial city of Turkey	BMI changes in the intervention group1 and intervention group2 vs the control group were 0.37 ± 1.08, 0.35 ± 1.13 vs 0.51 ± 0.98, which was significantly different (p = 0.000). There was no significant difference between the intervention groups (P = 0.847). the BMI change of the students in the control group was associated with the income level of the family (p = 0.005). This	NA

Reference	Adoption	Component & delivery method	Duration	Context	Outcome	Maintenance
		and seasonal fruits were sold in school canteen.			relationship was not shown in the intervention groups.	
Xu HQ, et al, 2017.	• Introduction of the intervention<br>1. Schools' classroom tutors and/or health educators attended training sessions on how to integrate the program into the school curriculum and to perform the activities. They received slides and videos about nutrition, childhood obesity, risk factors, health consequences, and prevention prepared by Chinese CDC.<br>2. Parents attended 2 workshops by nutrition professionals to encourage their children to have a healthy diet.<br>• Adoption of stakeholders<br>The school staff modelled the lessons to ensure that they understood the recommended techniques and strategies for implementation. No information on the extent of adoption from the stakeholders.	Group 1<br>1. ENE for students.<br>40-minute lecture X 6 sessions about healthy eating proportions of three meals, how to choose the beverage and snacks, reducing eating out and Western-style fast food. Cartoon pamphlets were provided.<br>2. Nutrition education for teachers and school staff<br>40-minute lecture X 4 times<br>3. Nutrition education for parents<br>40-minute lecture X 2 times<br>4. Provision of learning materials including a nutrition handbook, "Dietary Pagoda for Chinese people" posters displayed on classrooms' walls. Cartoon handbooks containing all of this information were distributed to all participants in the nutrition education group to help clarify the concepts presented in the lectures.<br>5. Regular monitoring of the nutritional quality of school lunch menu and suggestions for improvement	1 school year	Schools in the capital city of China	The overweight and obesity prevalence in the control group increased by 1.5% (p<0.001), while there was no significant change in the intervention group (0.2%, p=0.954). Compared with the control group, the comprehensive intervention effects could be found (BMI mean difference: -0.3 kg/m2 (-0.4, -0.2), p<0.001, BMI for age z score mean difference: -0.14 (-0.18, -0.11), p<0.001	NA

Reference	Adoption	Component & delivery method	Duration	Context	Outcome	Maintenance
		Group 2<br>Extra PA<br>10-minute of preferable PA (25-35.1 kcal) X 2 times/day for 5 days/week or the "happy 10"<br><br>Group 3<br>Group 1 and 2 were combined.				
Cao ZJ, Wang SM, Chen Y, 2013.	• Education Bureau and Institute of Education involvement established an administrative system (e.g., relevant rules and regulations), supervised school canteen staff, and provided funding for the project<br>• Research team organised trainings for school staff to integrate the intervention program into the school curriculum (teachers as agents at school)<br>Research team invited experts to conduct lectures for students and their parents (parents as agents at home)	1. Health education<br>- Nutrition classes (6 hrs) /term (obesity risk factors, health consequences, and obesity prevention)<br>- Obesity related information dissemination through school communication platform e.g., morning meetings, blackboard newspaper, brochures, seminars etc.<br>- Parent-school meeting every term and brochures<br>2. Dietary intervention<br>- Reduce fat content in school food and increase availability of fruit and vegetables<br>- Provide information about balanced diet principles and methods and instructions for parents to help children to have a healthy diet<br>3. Exercise intervention	33 months	Shanghai, China. Urban city. Highest prevalence of obesity among school-aged children. The increasing trend of childhood obesity gained great attention from all sectors.	The overall prevalence of overweight/obesity declined from 28.92% in 2011 to 24.77% in 2014, with a difference of 4.15% in the intervention group compared with a 0.03% decline (from 30.71% to 30.68%) in the control group. The odds of developing obesity among IG vs CG was 0.583 (0.428, 0.794), $p<0.001$, while of developing obesity & overweight of IG vs CG is 0.625 (0.493, 0.793), $p<0.001$. BMI z-scores of IG vs CG: overweight students $\beta = -0.030$ (-0.049, -0.011), 0.002 and obese students $\beta = -0.046$ (-0.072, -0.021), $p<0.001$.	NA

Reference	Adoption	Component & delivery method	Duration	Context	Outcome	Maintenance
		- At least 1 hr PA/day: 20-meter music shuttle run 2–3 times/wk and fun sports e.g. football, rope skipping<br>- Rope was provided to students to do rope skipping at home during school breaks (PA plan and form was provided for parents to monitor and record students' PA)				
Guo HT, Zeng XS, Zhuang QY, Zheng YB, Chen SR, 2015.	The intervention was conducted by investigators	1. <u>Nutritional education session</u> 1hr lecture/month & brochures to teach how to eat healthily (less calories & fat & more nutritious)<br>2. <u>Exercise session</u> 1-hr session to have at least 1hr medium or vigorus PA a day (reaching 70-80% maximum heart rate)<br>3. <u>Psychological intervention</u> 1-hr psychological education and consultation session/month<br>4. <u>Fun PA contest</u> 1-2 PA contests during school breaks<br>5. <u>Telephone</u> follow-up phone calls every 2 weeks	1 year	Shantou, a coastal city and special economic zone in China.	BMI (kg/m^2): IG baseline 22.18 ± 2.13, post intervention 22.95 ± 2.53 (p=0.002), CG baseline 22.05 ± 2.57 post intervention 23.16 ± 2.39 (p=0.013). BMI z score: IG baseline 1.56 ± 0.33 post intervention 1.47 ± 0.44 (p=0.036), CG baseline 1.45 ± 0.35 post intervention 1.48 ± 0.31 (p=0.617)	NA
Shofan Y, Kedar O, Branski D, Berry E, Wilschanski M, 2011.	The programme was led by the Braun School of Public Health in cooperation with the Ministry of Education. PA guideline for	1. <u>Nutrition education lessons</u><br>2. <u>Extra aerobic component PE</u> 2x (duration) PE (increase aerobic component by 50%. At baseline, the normal	2 years	School with 350 students, Israel	BMI (mean±SD (range)): IG baseline 17.9±3.9 (11.5–33), post intervention 18.7±4.6 (12–38), mean difference	

Reference	Adoption	Component & delivery method	Duration	Context	Outcome	Maintenance
	children was disseminated to schools to follow. Health professionals provided nutrition education to children ad encouraged parents to provide healthy diet to children.	physical education classes consisted of two lessons of 45min each of medium intensity training with an estimated aerobic component of 25%<br>3. Monthly meeting with parents<br>1hr session/month X 10 months (no detail of activities)			0.94±1.5 (-2.9–5.5). CG baseline 18.9±4.3 (13.4–33), post intervention 19.4±4.6 (13–31), mean difference 0.48±1.23 (-2–3.5).	
Lee GY, Choi YJ, 2016	Nursing students were trained (16hr) to mentor students. They were informed about their roles, therapeutic relationship, and effective communication.	1. Mentoring<br>1 nursing student mentored 2-3 students. Mentors contacted their mentees once a week to check their daily PA and eating habits. The mentors were evaluated and supervised by psychiatric nurses.<br>2. NE & PE<br>8 classes to provide knowledge about obesity, self assessment on BMI & calorie intake, healthy eating, healthy exercise, fun sport, self-motivation and aspiration.	10 weeks	Seoul Capital Area. Awareness of childhood obesity is low in Korea. Snacks provided in schools were mostly unhealthy.	BMI (mean±SD): IG baseline 25.3 (±3.22) post intervention 24.7 (±3.37), CG baseline 24.6 (±2.70) post intervention 24.5 (±1.86) (p< .0001).	NA
Li B, Pallan M, Liu WJ, Hemming K, Frew E, Lin R, et al, 2019	Recruited 5 programme teachers to coordinate and deliver the programme. The teachers were trained and linked with school staff and families. Programme's handbooks were provided to	1. NE for children<br>Individuals' behavioural goals setting, self monitoring, and supervision.<br>2. Interactive nutrition workshop for carers<br>- correcting common misperceptions about child	1 year	State-funded primary schools in Guangzhou, the largest and one of the most socioeconomically advanced cities in South China.	BMI z score was significantly lower in the intervention compared with the control group, MD = −0.13, 95% CI: −0.26 to 0.00, p = 0.048 in the baseline-adjusted model; MD = −0.13,	NA

Reference	Adoption	Component & delivery method	Duration	Context	Outcome	Maintenance
	intervention schools to guide the schools.	healthy weight and healthy behaviours,<br>- introducing practical parenting tips for encouraging healthy behavioural change in children<br>- Providing leaflets<br>3. <u>Healthy school food provision</u> Healthy school lunch goal setting among the programme's teachers and school food providers. Constructive evaluation on the food menus.<br>4. <u>PA promotion outside schools</u> Taster session to teach fun & active family games at home. PA homework assignment.<br>5. <u>PA promotion at school</u><br>- Situation analysis<br>- Monthly goals setting<br>- Action plan to meet the goal & regular evaluation & advice.<br><br>Programme was mainly delivered as planned.			95% CI: −0.26 to −0.01, p = 0.041 in the further-adjusted model	
Li YP, Hu XQ, Schouten EG, Liu AL, Du SM, Li LZ, et al, 2010	A half-day training session for teachers conducted by the staff of the National Institute for Nutrition and Food Safety, China CDC. This is to inform teachers about integrating the	<u>PA promotion</u><br>Teachers added extra PA activity twice daily using teaching materials: cards (to select activity), video demonstrations, tracking posters, and stickers (to track the progress). The 10-minute PA session (moderate or	1 year	Urban Beijing, China.	BMI increased by 0.56 kg/m2 (SD 1.15) in the intervention group and by 0.72 kg/m2 (SD 1.20) in the control group, with a mean difference of -0.15 kg/m2	After another year of follow up, compared to the control group, children in the intervention group had significantly lower

Reference	Adoption	Component & delivery method	Duration	Context	Outcome	Maintenance
	programme activities into school curriculum, childhood obesity and prevention.	vigorous intensity) consisted of move, cooldown, health message provision (caloric expenditure ranged from 60-70 kcal/day).			(95% CI: -0.28 to -0.02), p=0.03. BMI z score decreased by -0.05 (SD 0.44) in the intervention group but increased by 0.01 (SD 0.46) in the control group, with a mean difference of -0.07 (-0.13 to -0.01), p=0.03.	BMI mean difference between IG and CG (-0.13, -0.25 to -0.01), p=0.04. BMI z score mean difference between IG and CG (-0.05, -0.10 to -0.01), p=0.03.
Liu A, Hu X, Ma G, Cui Z, Pan Y, Chang S, et al, 2008	Happy 10 was initiated by the National Institute for Nutrition and Food Safety, Chinese Center for Disease Control and Prevention. No information available on how the programme was introduced to the schools.	PA Promotion<br>Teachers added extra 10-minute exercise at least once every school day. Posters and stickers were used to track the progress of each class.	8 months	urban Beijing, China.	BMI in IG: Boys Baseline 18.09, post intervention 18.95, change 0.86 (<0.05) Girls Baseline 18.63, post intervention 18.16 change -0.47 (<0.05).<br>BMI in CG: Boys baseline 17.96, post intervention 18.68, change 0.72 (<0.05). Girls baseline 16.42, post intervention 17.08, change 0.66 (<0.05). There was a significant difference in the change between the intervention school and control school after intervention among girls.	NA
Liu Z, Li Q, Maddison R, Mhurchu CN,	NA	1. School policies	1 year	Urban Beijing, China. Increasing ‘‘obesogenic’’	Mean BMI (kg/m2) and BMI Z-score were 18.57 (SD 3.60)	NA

Reference	Adoption	Component & delivery method	Duration	Context	Outcome	Maintenance
Jiang YN, Wei DM, et al, 2019.		Sugary drinks ban, water drinking campaign, healthy lunch, 60 MVPA/day,<br>2. Health education (diet and PA)<br>classes, posters, broadcast, website, teaching materials, diet & PA diaries, drawing contest.<br>3. PA promotion<br>>60 MVPA/day, sport equipment, sport club for overweight & obese.<br>4. Healthier school lunch<br>school lunch improvement<br><br>The quantity of intervention delivery was 80%–100% for intervention elements among most intervention schools. Variability exists for the quality of intervention delivery, which was highest for the sport club for children with overweight/obese.		environment. Among the school-based interventions in China, few have focused specifically on environmental strategies.	and 0.31 (1.19) in the intervention group and 18.46 (3.76) and 0.28 (1.23) in the control group. The model-adjusted group differences (intervention vs. control) in BMI and its Z-score at 12 months were 0.07 (95% CI: -0.16 to 0.31; $p = 0.54$) and 0.02 (95%CI: -0.08 to 0.11; $p = 0.73$). The estimated intraclass correlation coefficients were 0.04 and 0.05, respectively, consistent with the initial assumption. Obesity intervention vs control groups at 6 months (OR: 0.50, 95% CI: 0.26 to 0.96; $p < 0.05$).	
Habib-Mourad C, et al, 2020.	• Introduction of the intervention<br>1. Private schools were approached directly. Public schools were recruited by the MOE.<br>2. Investigators provided training to teachers.	1. NE for students<br>12 nutrition education interactive classes (10 to 15 min of discussion about the topic of the week followed by 30 min of games and/or food preparation) in the 1st year and 6 complementary activities in the 2nd year by teachers who had received a 'training of trainers	2 school years	Beirut, the capital of Lebanon	There were no changes in terms of BMI, BMI for age z score, and overweight and obesity prevalence at the end of intervention.	After one-year washout, the intervention group had a 52% reduced odds of being overweight/obese compared to students in control group. The intervention was

Reference	Adoption	Component & delivery method	Duration	Context	Outcome	Maintenance
	3. Investigators suggested school food chops to provide healthier food.	(ToT)' workshop on all program components.<br>2. <u>Parent meetings and health fairs</u><br>Showed examples of healthy meals, food recipes and provided pamphlets.<br><br>3. Provision of healthy food<br>Asked school shops to sell healthier food in school shops and asked parents to prepare healthy lunch boxes				scaled up and rolled out as Ajyal Salima–Healthy Kids, a mandatory component in the public schools' health curriculum.
Lin YC, et al, 2019	The intervention was conducted by research team to assure the consistency.	<u>NE & PE training course</u><br>The image of the astronaut to stimulate children to implement a healthy lifestyle. The 40-min class/weeks X 8 consecutive weeks by the research group. Online materials that can be easily integrated into math, science, and reading classes.<br>Water bottles and "space logs" were distributed to motivate the children to keep training and recording their physical activities and diet at school and at home.	8 weeks	Remote rural areas of Northern Taiwan	BMI (kg/m2) (mean (SD): IG baseline 19.51 (4.37), post intervention 19.54 (4.32), CG baseline 18.41 (3.34), post intervention 18.62 (3.26).<br>BMI z score (kg/m2) (mean (SD): IG baseline 0.72 (1.39), post intervention 0.71 (1.31), CG baseline 0.54 (1.09), post intervention 0.60 (1.04). No significant changes.	
Meng LP, et al, 2013.	Investigators provided nutrition & health education to students, teachers, parents, health workers. Teachers were trained to provide PE.	1. <u>Nutrition education for children</u><br>6 nutrition and health class<br>2. <u>Training course for parent</u><br>2 times for the parents	1 school year	Beijing, Shanghai, Chongqing, Guangzhou, Jinan and Harbin	BMI and BAZ increment was 0.65 kg/m2 (SE 0.09) and 0.01 (SE 0.11) in the combined intervention, respectively,	NA

Reference	Adoption	Component & delivery method	Duration	Context	Outcome	Maintenance
		3. 4 times for teachers and health workers.<br>4. The menu for students of school lunch cafeteria was evaluated periodically and specific nutrition improvement was suggested accordingly PA: In each school day, the students were conducted "Happy 10" led by teachers to do a 10-minute segment moderate intensity, age- and space-appropriate exercises. The form of exercises was game, dance or rhythmic gymnastics. Students were also encouraged to develop more forms of exercises they like. Furthermore, education about physical activity was provided to students, parents, health workers and teachers. Each student attended the "Happy 10" 10 minutes for once, twice a day or 20 minutes for each time, once a day. Nu ed + PA			significantly lower than that in its' control group (0.82±0.09 for BMI, 0.10±0.11 for BAZ). No significant differences were found neither in BMI nor in BAZ change between the PA intervention and its' control, which is the same case in the nutrition intervention	
Bhave S, et al, 2016.	• Introduction of the intervention<br>1. Meetings were held to inform families and teachers about the study.<br>2. Investigators trained teachers to deliver the intervention.	1.Extra PA<br>6 PA sessions/week, making PE a 'scoring' subject that contributed to the children's academic marks, engage the children in daily yoga-based breathing exercises ('pranayam'), and offering attractive physical activity	5 years	A symbiosis school in Pune has a strong academic reputation and most students had high socio-economic status. The school provided paid	No significant changes were found.	NA

Reference	Adoption	Component & delivery method	Duration	Context	Outcome	Maintenance
	3. Investigators encouraged kitchen staff to provide healthy food.<br>4. Parents were trained and asked to prepare healthier lunch boxes.<br>• <u>Adoption of stakeholders</u><br>No information on the extent of adoption from the stakeholders.	sessions (e.g., 'Bollywood dancing') during holidays<br><u>2. Provision of healthy food</u><br>Nutritionists worked with kitchen staff to provide healthier lunch with more fruit and vegetables and ban fast-food sellers from outside the school gates.<br><u>3. NE</u><br>1 hr session/week teaching about the importance of diet, healthy eating, active lifestyle and integrate nutrition into science classes.		lunch for students, and two compulsory 40-minute physical education (PE) periods per week. Outdoor activities are constrained by a small playground.		
Liang Y, Lau PWC, Jiang YN, Maddison R, 2020	The investigators conducted the intervention.	<u>PA Promotion</u><br>After-school extracurricular 1-hr PA session (10–15 minutes warm-up, followed by the active video game (AVG game)) X 8 weeks using two game consoles-Xbox 360 Kinect™. Trained researchers led the AVG classes. The game required both upper and lower body movements.	8 weeks	Hong Kong-China. Limited outdoor space in primary schools (2 m^2/student) and constraints of outdoor programs (i.e., safety concern, air pollution, and inclement weather).	BMI z score (mean (SD)) in the IG at baseline was 0.4 (1.4), at post intervention 0.4 (1.3). Mean difference (IG mean difference – CG mean difference) was 0 (−0.1, 0.1), $p = 0.42$.	NA
Toruner EK, et al, 2015.	The investigators conducted nutrition education.	4 training sessions over 3 months. Each training session lasted 1 class hour (an average of 40 minutes). The sessions included the presentation of information on (a) healthy life, (b) nutrition, (c) sedentary lifestyle, and (d) physical exercise through lectures, discussions, and short messages.	3 months	Turkey	No significant difference was observed in the initial and final BMI of the intervention Group.	NA

Reference	Adoption	Component & delivery method	Duration	Context	Outcome	Maintenance
		Before the 2nd, 3rd, and 4th training sessions, short messages about the previous training session(s) were used to prompt the children. Teaching methods included playing games, videos, and show and tell. group playing was performed with red, yellow,<br>and green traffic lights for healthy and unhealthy foods; and 2 short cartoons (2 to 3 minutes) were presented. Teachers joined the sessions with the students. Brochures were sent to families.				
Wang JJ, et al, 2015.	Medical research postgraduate students implemented the intervention with the help of a physical trainer.	Used social cognitive theory.<br>1. PA<br>Extra exercise sessions were prescribed.<br>2. NE<br>Lectures about diet and PA were provided during the parental meeting (Education materials and content were delivered to the parents without attendance<br>by the mail and phone)<br>3. Diet prescription and monitoring<br>Normative feedback & individual feedback were given to students (in person) and parents(telephone)). Gifts were awarded to students who achieved their goals.	1 year	China	Positive but non-significant adjusted changes in body mass index and waist circumferences were observed.	NA

Reference	Adoption	Component & delivery method	Duration	Context	Outcome	Maintenance
Xu F, Ware RS, Leslie E, Tse LA, Wang ZY, Li JQ, et al, 2015.	The NE and PE were integrated into the current health eductation. Healthy diet and PA promotion messages were delivered to parents through regular parents meetings. All teachers were trained and provided with the same teaching materials to present for students.	1. NE and NE curriculum<br>30-minute lesson/month X 8 months (delivered by teachers). The content was about healthy diet, active lifestyle, and tips to a healthy lifestyle. The classes were monitored by the research team.<br>2. School environment support<br>Monthly updated posters made by students presented at classroom, gymnasium, playground, and cafeteria.<br>3. Family involvement<br>Carers meeting twice a term run by researchers to provide knowledge about childhood obesity, healthy diet, and active lifestyle. Interactive assignment about high-dense energy home food and BMI assessment was offered.<br>4. Fun programme/events<br>a) presentation competition (tell their stories by drawing). A stage drama of the story wining the prize.<br>b) no unhealthy snack week.<br>c) no TV week.<br>d) No soft drink week.	1 year	An urban district of Nanjing. Health education is compulsory for all primary and high schools. The first priority of family and schools is academic performance.	Mean differences of BMI (SD) among IG vs CG: -0.32±1.36 vs. -0.29±1.40, p = 0.09.	NA
Toruner EK, Savaser S, 2010.	NA	1. NE for children<br>1-2 hr(s) training session x 7 sessions in 2.5 months about self-recognition, self-expression, healthy nutrition, physical exercises, the negative effects of a sedentary life, and goal setting	13 months	Ankara capital city of Turkey.	No statistically significant difference was detected between the first and second BMI measurements in both the intervention and the control	

Reference	Adoption	Component & delivery method	Duration	Context	Outcome	Maintenance
		based on Social Cognitive Theory.<br><br>2. Training session for parents Two sessions to increase awareness of childhood obesity.<br>3. Consultation for parents 30-50 minutes session.<br><br>This is to improve eating and PA behaviours at 3 levels: 1. Psychological -to improve intention, perception of one's own capacity, and goal setting, 2.Behavioural-healthy eating and active lifestyle promotion 3. Environmental- parents to encourage healthy eating and PA at home.			groups. BMI (kg/m2) (mean (SD)): IG at baseline 23.1 (2.0), at post intervention 22.5 (1.8). CG at baseline 23.2 (2.5), at post intervention 23.5 (2.4).	
